# Mobile Device Use in Children Aged 0–11 Years—Surveys in the Netherlands and Spain

**DOI:** 10.1002/bem.70072

**Published:** 2026-10-21

**Authors:** Silvia Ariccio, Dora Cserbik, Patricia de Llobet Viladoms, Marthe Knijff, Bastien Tavner, Laura Draetta, Elisabeth Cardis, Monica Guxens, Anke Huss

**Affiliations:** ^1^ Institute for Risk Assessment Sciences Utrecht University the Netherlands; ^2^ ISGlobal Barcelona Spain; ^3^ Spanish Consortium for Research on Epidemiology and Public Health (CIBERESP) Instituto de Salud Carlos III Madrid Spain; ^4^ C.I.U.S Healthcare Innovation Lab Nice France; ^5^ i3 (CNRS‐Telecom Paris) Institut Polytechnique de Paris Paris France; ^6^ ICREA Barcelona Spain; ^7^ Universitat Pompeu Fabra Barcelona Spain; ^8^ Department of Child and Adolescent Psychiatry/Psychology, Erasmus MC University Medical Centre Rotterdam The Netherlands

**Keywords:** activities, calling, devices, EMF, exposure, streaming, toddlers, young children

## Abstract

While relevant for public health policy‐making, little information is currently available about children's usage of mobile devices and on the activities performed with these devices. This paper presents results of two surveys conducted in the Netherlands and one in Spain about the activities children perform on mobile devices (Netherlands: 0–11 years, Spain: 3–11 years). Parents reported in questionnaires the type and duration of activities performed by children with different device types (smartphone, tablet, laptop). Descriptive analyses stratified by different socio‐demographic variables are reported. Cluster analyses were conducted to explore patterns of usage. Results show that even young children (0–2 years old) perform some activity, usually calling or video streaming. The number and the duration of activities were highest among the older children (9–11 years). The kind of activities children perform changes with age, with some activities emerging early, such as video streaming, and others later, like videogaming. Cluster analysis shows that gender differences may already exist in the 0–2 years old range, with girls performing more calling activities than boys. These results can inform future studies and policy making on long‐term effects of mobile devices exposure and on psychological and mental health. Bioelectromagnetics. 00:00–00, 2026. © 2026 Bioelectromagnetics Society.

## Highlights

Surveys were conducted in the Netherlands and in Spain about the activities of 0–11 years old children on mobile devices. The kind of activities changes with age, older children perform more activities. Even 0–2 years old children perform some activity, gender differences may already exist in that age range. Bioelectromagnetics.

## 1 Introduction

A considerable body of research has focused on quantifying radiofrequency electromagnetic field (RF‐EMF) exposure and on investigating its short‐ and long‐term health consequences (Bosch‐Capblanch et al. 2024; Röösli et al. 2021). In adolescents and adults, a substantial proportion of RF‐EMF exposure stems from personal mobile device use (Eeftens et al. 2023). Some specific types of device use, such as calling with the device held next to the ear, are known to result in substantial exposure to the brain, which has been a particular focus of research in the past, due to its potential relevance to health. However, empirical data on cross‐country device use in children, especially those of very young age, remain limited (see Department of Education 2024 for a recent UK study limited to screentime). Moreover, studies mostly focus on duration of usage and sometimes on device position during usage, but little research has been done on the specific activities people conduct during usage (see Stamets et al. 2025 for a rare example) while usage patterns are a key determinant of RF‐EMF exposure. The aim of this study was thus to explore mobile device use of children aged 0 to 11 years and their related exposure‐relevant usage types.

## 2 Methods

Within the framework of the EXPOENFANTS project (approved by the ethical committee CEIm—Parc de Salut MAR No. 2022/10411, on 07/06/2022), epidemiological and sociological questionnaires were developed to identify key sources of exposure, device use, and technologies, as well as the sociological factors influencing usage patterns across different ages.

Data collection consisted of two surveys employed to collect three data sets, two in the Netherlands and one in Spain. In all cases, the target participants were parents, who, after answering a few questions about their age and their educational level, were asked to report on the behavior of their child. If parents had more than one child, they were asked to report for their youngest child. Responses were collected anonymously after obtaining informed consent. In the Netherlands, the first data set was collected using an online survey created and administered via Qualtrics (Provo, UT; www.qualtrics.com), In this wave, targeted participants were parents with at least one child in the age group between 0 and 11 years of age. They were invited via social media campaigns of the Utrecht University communication channels, including the university website, intranet, and the LinkedIn and Facebook pages. The survey was conducted between July and August 2024. Although the targeted population size was a minimum of 250 parents, a total of 740 parents completed the questionnaire. We will refer to this data set as NL‐QUALT.

The second wave of data collection was conducted in the Netherlands and Spain through the private survey company Cint (CINT, www.cint.com). Participants were recruited from Cint's registered panel members between September and November 2024. Recruitment followed a stratified sampling plan with age‐specific quotas (targeting ~200–300 participants per age bracket). While child gender followed a natural fallout distribution, parental education and level of urbanization of the place of residence were actively monitored to ensure balanced representation, resulting in an overall participation rate of 24%. Eligibility criteria included parents with children aged 1–11 years in the Netherlands (*N* total = 668), and 3–13 years in Spain (*N* total = 612).

For the Spanish sample, only participants with children aged 3–11 years were included in this paper (*N* = 406). We will refer to these data sets as NL‐CINT and SP‐CINT, respectively.

Table 1 summarizes the main characteristics of the three data sets.

**Table 1:** Main characteristics of the three data sets.

	NL‐QUALT	NL‐CINT	SP‐CINT
Country	Netherlands	Netherlands	Spain
Recruitment strategy	Media campaign	CINT	CINT
Time period of data collection	July–August 2024	September–November 2024	September–November 2024
Age range	0–11	1–11	3–13
Main survey differences	No device‐level activity information	Device‐level activity information	Device‐level activity information

Questionnaires were based or adapted on previous studies, or they were previously piloted by volunteers. All questionnaires included the following sections: (1) sociodemographic variables of both children and parents (age, sex, education, country of residence, and family composition); (2) the duration and use of mobile devices (phone, tablet, and laptop). For the scope of this paper these activities included phone‐calling with a cordless phone, phone‐calling with a mobile phone (via mobile network), calling via an app, video calling via an app, streaming audio contents, streaming video contents, streaming audio contents, and gaming. However, some differences between the questionnaires existed. Notably, the questions about performance of each activity and of its duration were phrased differently in the Qualtrics and the CINT versions of the questionnaire. In the NL‐QUALT survey, for each activity, it was asked if and how many minutes per week the child would perform it. In the CINT data sets (NL and SP), for each activity, it was asked to report for each device if and how many minutes and/or hours (reported per day or per week) the child would perform the activity: on the smartphone, on the tablet, and on the laptop. Samples of the questionnaires are available in SM1 Questionnaire samples.

## 3 Statistical Analyses

For each variable, descriptive statistics were reported, notably frequency for categorical and ordinal variables, and median, 25th and 75th percentile (interquartile range (IQR)) for numerical variables. Results are presented by children's age group (“0–2”, “3–5”, “6–8”, “9–11” years). These age groups have been defined to ease the presentation of results while facilitating inter‐group comparisons.

Duration of all activities had very different median and mean scores in the NL‐QUALT vs. CINT surveys, likely because of the different formulation of the questions. To account for these differences, analyses were run separately by data set. For each activity duration, extreme outliers were detected (defined as Q1 − 3 × IQR or Q3 + 3× IQR, as in Tukey 1977) and replaced by the median value of the variable. A total of 62 outliers were identified in the NL‐QUALT data set, 75 in the NL‐CINT data set, and 10 in the SP‐CINT data set. Due to its unusual distribution (IQR = 0), no outlier was identified with this method in the video streaming duration variable in the SP‐CINT data set. A manual outlier detection method was thus employed and identified a single outlier which was replaced with the median (minutes per week = 5040).

Additional descriptive analyses were conducted to evaluate whether the age trends observed vary by level of the socio‐demographic variables: child's sex, level of urbanization of the place of residence, parent's age and level of education of parent filling in the survey. Linear regression analysis was employed to test associations between age and number of activities.

Cluster analysis was employed to identify clusters in the data sets that were otherwise not directly visible by inspecting the data stratification tables (see supporting Information S1). Since cluster analysis, as run by the library “cluster” does not accept any missing data in the data set, before running the cluster analysis, missing data were replaced. As a first step, to maintain coherence in the data sets, if the duration of an activity was NA and the activity had been indicated as not performed, its duration was set to “0”. After this preliminary procedure, NA were replaced with median (in the case of numeric variables, notably durations) and mode (in the case of categorical variables, notably activity performance). We then ran hierarchical cluster analyses per data set, using Gower distance, to account for both numeric and categorical variables. The definition of the number of clusters was done on the basis of visual hierarchical tree analysis. Median and mode scores were then used to characterize the clusters. Each data set was considered as a reference comparison group for clusters emerging in that data set. Thus, when the mode of the cluster's socio‐demographic variables was different from the one of the whole data set, that value was reported to describe the cluster. Most represented age group in each cluster was always reported for ease of interpretability.

Finally, we performed a sensitivity analysis to assess if parental rules on children's use translated into different usage patterns. Four questions asked parents if they had defined rules for the type of activities, the moment of usage, the length of usage, and the type of device (1 = No rules, 2 = Flexible rules, 3 = Strict rules). Exploratory factor analysis indicated that the items were all fitting on the same factor, which had good reliability (Cronbach's *α* = 0.85 in NL‐QUALT, 0.80 in NL‐CINT, and 0.80 in SP‐CINT).

Here, we tested the interaction effect of the strictness of rules and children's age on the duration of usage for the activities considered by this study that specifically required the use of a screen: video calling, video streaming, and videogaming.

## 4 Results

### 4.1 Population Description

A total of 1814 completed surveys were collected, 1408 in the NL, and 406 in Spain (Table 2). In the Netherlands, 668 parents filled in the NL‐CINT survey, and 740 parents the Qualtrics online questionnaire. The Spanish survey (SP‐CINT) was completed by 406 participants. Median age was 4 years in NL‐QUALT, 7 years in NL‐CINT, and 6 years in SP‐CINT survey, respectively. Parental education and level of urbanization of the place of residence differed substantially between NL‐QUALT and the other two surveys, with 90% of parents reporting university education, compared to 50%–60% in the CINT surveys and only 14% reporting living in a city compared to 40%–50% in the CINT surveys.

**Table 2:** Description of the study population.

Variables	NL‐QUALT *N* = 740	NL‐CINT *N* = 668	SP‐CINT *N* = 406
Child's age	4.0 (2.0, 7.0)	7.0 (4.0, 10.0)	6.0 (5.0, 9.0)
Child's sex			
Girl	350 (47%)	319 (48%)	180 (44.3%)
Boy	388 (52%)	349 (52%)	226 (55.7%)
Other	2 (0.3%)	0 (0%)	0 (0%)
Parent's age at birth	33.0 (31.0, 36.0)	29.0 (25.5, 33.0)	31.0 (27.0, 36.0)
Parent's education			
No‐University	71 (9.6%)	324 (49%)	180 (44.3%)
University	667 (90%)	344 (51%)	226 (55.7%)
Level of urbanisation			
Village	301 (41%)	120 (18%)	51 (12.6%)
Town	333 (45%)	274 (41%)	148 (36.5%)
City	103 (14%)	274 (41%)	207 (51%)
Median (IQR); *n* (%)			

*Note:* Interquartile range (IQR): 25th percentile, 75th percentile.

### 4.2 General Descriptives of the Different Data sets

Overall, participants who completed the NL‐CINT and SP‐CINT questionnaires reported consistently substantially longer durations of activities compared to participants in the NL‐QUALT survey. In addition, participants in NL‐CINT reported a higher number of total activities, with a median of three activities, compared with a median of 2 activities in both the NL‐QUALT and SP‐CINT data sets.

Tables 3, 4, 5 show general characteristics. With advancing age, more children engage in a variety of activities both in The Netherlands and in Spain. The estimated linear effect of age on number of activities is 0.25 in the NL‐QUAL, 0.15 in the NL‐CINT data set, and 0.10 in SP‐CINT (all *p* < 0.01), after controlling for sex effects. Overall, such a progression suggests a gradual diversification of uses, from more passive activities such as video streaming to more interactive and socially mediated uses such as calling and gaming.

**Table 3:** Descriptives of mobile device use in the NL‐QUALT data set.

Activities	Overall *N* = 740	0–2 *n* = 213	3–5 *n* = 225	6–8 *n* = 175	9–11 *n* = 127
Phone calling (yes vs. no)^1^	250 (34%)	64 (30%)	72 (32%)	47 (27%)	67 (53%)
minutes per week^2^	10 (5, 10)	7 (5, 15)	10 (5, 15)	10 (4, 10)	10 (5, 10)
App calling (yes vs. no) ^1^	108 (16%)	13 (6.4%)	31 (14%)	19 (12%)	45 (39%)
minutes per week^2^	5.0 (3.0, 10.0)	5.0 (3.0, 10.0)	5.0 (2.0, 10.0)	5.0 (2.0, 10.0)	5.0 (5.0, 15.0)
Video calling (yes vs. no) ^1^	209 (32%)	51 (26%)	85 (42%)	27 (17%)	46 (44%)
minutes per week^2^	10 (5, 15)	10 (5, 25)	10 (5, 10)	10 (5, 15)	10 (5, 20)
Video streaming (yes vs. no) ^1^	403 (62%)	61 (32%)	139 (71%)	120 (75%)	83 (83%)
minutes per week^2^	120 (30, 300)	30 (15, 105)	70 (30, 200)	200 (60, 400)	210 (60, 420)
Audio streaming (yes vs. no) ^1^	208 (33%)	42 (23%)	52 (27%)	60 (39%)	54 (55%)
minutes per week^2^	60 (30, 90)	30 (10, 60)	60 (30, 100)	60 (30, 60)	60 (30, 120)
Gaming (yes vs. no) ^1^	213 (34%)	2 (1.1%)	42 (22%)	89 (58%)	80 (88%)
minutes per week^2^	100 (30, 210)	35 (10, 60)	30 (20, 60)	60 (45, 180)	190 (100, 300)
Cordless phone calling (yes vs. no)^1^	13 (2.1%)	1 (0.6%)	1 (0.5%)	4 (2.6%)	7 (8.0%)
minutes per week^2^	2.3 (0.0, 5.0)	0.0 (0.0, 2.5)	10.0 (0.0, 20.0)	1.5 (0.5, 2.5)	5.0 (0.0, 5.0)

*Note:* ^1^“Does your child use a mobile device, at least once per week, for…”; *n* and % of children whose parents reported this type of use, ^2^ “How many minutes per week does the child perform this activity on a smartphone/tablet/laptop?” (median number of minutes, IQR).

**Table 4:** Descriptives of mobile device use in the NL‐CINT data set.

Variables	Overall *N* = 668	0–2 *n* = 110	3–5 *n* = 172	6–8 *n* = 173	9–11 *n* = 213
Phone calling?^1^	403 (60%)	44 (40%)	86 (50%)	102 (59%)	171 (80%)
minutes per week^2^	120 (44, 420)	60 (20, 420)	70 (30, 190)	105 (50, 420)	120 (60, 420)
App calling? ^1^	335 (51%)	38 (35%)	64 (37%)	96 (56%)	137 (67%)
minutes per week^2^	105 (39, 270)	98 (35, 420)	85 (33, 420)	105 (30, 420)	105 (60, 210)
Video calling? ^1^	339 (52%)	50 (46%)	80 (47%)	87 (51%)	122 (60%)
minutes per week^2^	70 (30, 210)	70 (20, 122)	60 (15, 105)	60 (30, 210)	120 (60, 360)
Video streaming? ^1^	402 (62%)	52 (48%)	121 (71%)	110 (65%)	119 (58%)
minutes per week^2^	420 (210, 840)	420 (210, 840)	420 (140, 600)	420 (210, 735)	420 (300, 840)
Audio streaming? ^1^	186 (29%)	29 (27%)	48 (28%)	46 (27%)	63 (31%)
minutes per week^2^	240 (70, 525)	240 (150, 480)	240 (60, 630)	113 (60, 504)	240 (120, 630)
Gaming? ^1^	302 (47%)	21 (19%)	61 (37%)	104 (62%)	116 (58%)
minutes per week^2^	420 (165, 480)	210 (60, 480)	315 (120, 420)	420 (140, 480)	420 (210, 840)
Cordless phone calling? ^1^	72 (11%)	11 (10%)	22 (13%)	19 (12%)	20 (10%)
minutes per week^2^	121 (33, 420)	420 (60, 504)	60 (20, 270)	140 (30, 420)	122 (60, 350)

*Note:* ^1^“Does your child use a mobile device, at least once per week, for…”; *n* and % of children whose parents reported this type of use, ^2^ “How long does the child perform this activity on a smartphone/tablet/laptop?” (median number of minutes, IQR).

**Table 5:** Descriptives of mobile device use in the SP‐CINT data set.

Variables	Overall *N* = 406	3–5 *N* = 154	6–8 *N* = 118	9–11 *N* = 134
Phone calling?^1^	184 (45%)	57 (37%)	55 (47%)	72 (54%)
minutes per week^2^	140 (35, 210)	140 (70, 210)	105 (14, 210)	140 (32, 210)
App calling? ^1^	163 (40%)	47 (31%)	48 (41%)	68 (51%)
minutes per week^2^	210 (105, 210)	210 (88, 210)	210 (70, 210)	210 (140, 214)
Video calling? ^1^	174 (43%)	69 (45%)	50 (42%)	55 (41%)
minutes per week^2^	175 (105, 210)	140 (70, 210)	175 (105, 210)	210 (140, 210)
Video streaming? ^1^	255 (63%)	105 (68%)	70 (59%)	80 (60%)
minutes per week^2^	210 (210, 210)	210 (210, 210)	210 (210, 210)	210 (140, 210)
Audio streaming? ^1^	109 (27%)	37 (24%)	27 (23%)	45 (34%)
minutes per week^2^	210 (105, 210)	210 (70, 315)	210 (210, 210)	140 (105, 210)
Gaming? ^1^	172 (42%)	48 (31%)	50 (42%)	74 (55%)
minutes per week^2^	210 (105, 210)	210 (140, 210)	210 (105, 210)	175 (105, 210)
Cordless phone calling? ^1^	49 (12%)	19 (12%)	13 (11%)	17 (13%)
minutes per week^2^	105 (35, 210)	70 (11, 210)	158 (53, 263)	105 (53, 175)

*Note:* ^1^“Does your child use a mobile device, at least once per week, for…”; *n* and % of children whose parents reported this type of use, ^2^ “How long does the child preform this activity on a smartphone/tablet/laptop?” (median number of minutes, IQR).

Looking at the different age ranges, it can be noticed that 30%–40% of the very young children (0–2 year olds) used mobile devices for phone calls, and 26%–46% for video calling, with median duration of use as high as 60 min per week for phone calls and 70 min per week for video calls in the NL‐CINT survey. Other uses are also frequently reported in this age group, particularly in NL‐CINT, with median reported use of 210 min per week or more for video streaming, audio streaming, and gaming. Among 3–5 year olds, the number of activities seems to slightly increase, with especially video streaming becoming a common activity (> 65% in all three samples). In the 6–8 year old group, videogaming seems to gain popularity, with an increase of ≥ 25 percentage points in the Dutch data sets and an increase of 11 percentage points in the SP‐CINT data set. The other activities do not have a clear age trend, some becoming more common and other ones being stable or reducing a little bit their popularity. In the 9–12 year age range, there is a general increase in the variety of activities, with almost all activities having their highest diffusion in this age‐range, in all data sets. Cordless phone usage was reported to occur rarely: the maximum reported usage in any of the samples was 13%. Overall, calling‐related activities and video streaming were the first ones to occur, followed by gaming activities, mostly from 3 years old, and even more from 6 years old.

In general, the duration of activities increases with age in the Dutch data sets. This is particularly visible for some specific activity‐data set combinations, such as gaming in both Dutch data sets and phone calling in NL‐CINT. Most activities in the SP‐CINT data set do not show such increases in duration, except video calling and cordless phone calling.

### 4.3 Stratified Descriptives

Results stratified by child's age and (i) parent's age at child's birth, (ii) parent's educational level, (iii) level of urbanization of the place of residence, and (iv) child' sex are shown in the Supporting Information (see SM2 Stratified tables, Tables A1–D3).

Regarding the frequency of the various activities, a few differences between data sets emerge. When looking at sex differences, some differences emerge between the Dutch and the Spanish data sets, with video calling been more common among girls in the Dutch data sets, notably in NL‐CINT (47% vs. 57%), and among boys in SP‐CINT (46% vs. 39%). Differences between the two countries are also observed when looking at children's age in combination with parent's age: video streaming is positively associated to parent's age in the NL‐QUALT data set (from 52% among children with parents above 40 years old to 76% among children with parents below 30 years old), but it is negatively associated to it in the SP‐CINT data set (from 72% among children with parents younger than 30 years old to 50% among children with parents). When comparing children according to the level of education of their parent, a general trend emerges in the NL surveys with children of highly educated parents doing fewer activities than children of less highly educated parents. This is especially visible in the NL‐QUALT data set with app calling (30% vs. 14%) and gaming (49% vs. 33%). Such a trend is not clearly visible in the Spanish data set. When comparing children living in areas with different levels of urbanisation, a trend emerges in the CINT data sets, with children living in urban areas seemingly doing more call activities than children living in rural areas, for example, in the NL‐CINT children in urban areas do more phone calls (65%) than children in rural areas (48%) and in SP‐CINT children in urban areas do more app calls (46%) and video calls (47%) than children in rural areas (29% and 35%). Gaming instead has a different trend in the two countries, increasing with more rural areas in NL‐CINT (38% to 53%) and decreasing with more rural areas in SP‐CINT (46% to 25%).

Looking specifically at activity durations, a few trends emerge. In NL‐QUALT, girls spend more time on average doing calling activities (e.g., 15 min of median video calling for 0–2 years old), while boys tend to do more video streaming (with a median of 200 min among 9–11 years old). No clear trends emerged in the other data sets. Moreover, the NL‐QUALT data set, and partly also the NL‐CINT data set, show a trend regarding parents' education with parents' who have a university‐level education reporting their children do shorter video streaming and gaming (and also some calling activities) than children of parents with lower educational level. For instance, in NL‐QUALT children in the age group 3–5 years of parents with no university education stream videos for a median of 190 min a week, while those with university‐level educated parents only do this activity for a median of 60 min a week. In both cases, this trend seems to disappear as children grow older: Among older children, durations between children of educated and less educated parents even out, or slightly reverse. This trend is not clearly visible in SP‐CINT. As a last emerging trend, in the NL‐QUALT data set children living in less urban areas seem to be spending more time video streaming compared to children living in more urban area. For instance, children in the age group of 0–2 years and living in cities were reported to watch a median of 15 min of video streaming per week, while children in rural villages were streaming videos for a median of 60 min per week. This pattern is not visible in the other data sets.

### 4.4 Cluster Analyses

In both the NL‐QUALT data set and in the NL‐CINT data set, four clusters emerge, while five emerge in the SP‐CINT data set. See Supporting Information S1: SM3 Cluster descriptions for cluster characteristics (Tables E1–E3) and hierarchical trees (Figures F1–F3). Table 6 summarizes the clusters that emerged, organized by age groups and data set.

**Table 6:** Summary of the emerged clusters and their description, organized by the data set they belong to and their most represented age group.

Age groups	NL‐QUALT	NL‐CINT	SP‐CINT
0–2	NL_Q_0‐2a: mostly doing one activity, video streaming. NL_Q_0‐2b: notably girls, doing phone calling on top of video streaming.	NL_C_1‐2^a^: mostly doing one activity, video streaming.	
3–5		NL_C_3‐5: mostly girls, living in towns, with parents not highly educated, and older than the average, performing mostly two activities, i.e., video streaming and gaming	SP_C_3‐5a: with parents without university education, living in towns, performing one activity, namely video streaming. SP_C_3‐5b: with parents aged < 30, performing video streaming, but also phone call, app call, and videocall activities.
6–8	NL_Q_6‐8: mostly doing two activities, namely video streaming and gaming.		
9–11	NL_Q_9‐11: mostly doing six activities, namely phone calling, app calling, video calling, video streaming, audio streaming, and gaming.	NL_C_9‐11a: mostly girls, living in towns, with parents not highly educated, conducting three activities, namely phone calling, app calling, and video calling NL_C_9‐11b: conducting six activities for long durations, namely phone calling, app calling, video calling, video streaming, audio streaming, and video gaming.	SP_C_9‐11a: not engaging in any activity. SP_C_9‐11b: mostly boys, engaging in all considered activities, except cordless use, namely: phone calling, app calling, video calling, video streaming, audio streaming, and gaming. SP_C _9–11c: living in towns, with not highly educated parents, performing all activities except cordless calling and audio‐streaming.

*Note:* cluster names are composed by a short version of the data sets' name (“NL_Q” for NL‐QUALT, “NL_C” for NL‐CINT, and “SP_C” for SP‐CINT), followed by the typical age range of the cluster (“0–2”, “3–5”, “6–8”, “9–11”). When more than one cluster had the same typical age range in a data set, letters “a” and “b” were added to the name to distinguish them.

^a^ We know from the data collection that the NL‐CINT data set only included children from at least 1 yo, so the label has been adjusted to be consistent with the data.

Notably, results show that video streaming is the first common activity children use mobile devices for in all three samples, and even before 2 years old. Among 3–8 year olds, videogaming emerges as a new common activity within the Dutch samples, and, overall, videogaming and video streaming are the two recurrent common activities in this age group. Some children in the Spanish sample are also involved in calling‐related activities. Among 9–11 year olds, many clusters emerge, showing, on the one hand, a broad trend to start conducting all the activities; on the other hand, some children focus on calling activities (NL‐CINT sample) or show even no usage of mobile devices at all (SP‐CINT sample). A general trend shows that girls appear to be more involved in calling‐related activities, a pattern that becomes visible as early as ages 0–2 in the Dutch samples (see clusters NL_Q_0‐2b and NL_C_9‐11a). Other differences between clusters are associated with different levels of urbanisation, parents' age and parents' education, but overall no clear patterns are detected. Notably, cordless phone call use is never a common activity in our clusters.

### 4.5 Parental Rules

Our sensitivity analysis indicated a small yet statistically significant negative interaction effect between the strictness of parental rules and children's age on the duration of video streaming and videocalling in the NL‐QUAL population. Specifically, stricter rules were associated with shorter durations of these activities among older children only (more than 9 years old for videocalling and more than 5 years old for videostreaming). Conversely, the interaction was positive for videostreaming use, suggesting that stricter rules are only imposed when children already use these devices, and that the strictness becomes relevant in shaping usage practices only from 5 years onwards. No significant effects were found for videogaming, and none of the effects were significant in the NL‐CINT nor in SP‐CINT data sets. The results of these analyses can be found in SM4 Table G.

### 4.6 Discussion

#### 4.6.1 Summary of Results and Connections with Existing Literature

We explored usage of mobile devices across age groups in young Dutch and Spanish children. Overall, it can be noticed that even very young children (0–2 years old in the Dutch data sets, data for this age group were not available for Spain) performed some activity, usually related to calling or video streaming. There was consistency between the surveys that the number of activities is positively associated with age. The kind of activities children perform also changes with age, with some activities emerging early such as video streaming (emerging already before 3 years old in the Dutch samples), and others later, such as gaming (only becoming common from 3 or even 6 years of age).

Our study is in line with a study performed in four European Countries (UK, Luxemburg, Greece, and Malta) by Palaiologou (2016) which reports children under 3 years old to be already used to video streaming and half of them getting used to video‐playing before 6 years old. A Turkish study from 2014 had found both video streaming and gaming to be common among 3–6 year olds (Ata‐Aktürk 2014). Notably these studies collected data before smartphones were spread near‐ubiquitously, and, accordingly, these activities were usually performed on the TV or on the computer. In these older studies call activities were usually not considered since they were done with different devices, namely traditional landline phones (hard‐wired or cordless) or old mobile phones. Conversely, the low amount of cordless phone usage in our samples highlights how currently mobile device communication largely replaces traditional landline phone use.

Overall, results show age‐related progression from singular, more passive use in early childhood (primarily video streaming, later also gaming) to diversified digital engagement in later childhood, hinting that children may use parents' devices during their first years of life before they eventually have their own devices. This is in line with other European studies showing that smartphone ownership usually starts between 8 and 12 years of age (Albacete‐Maza et al. 2025; Cerimoniale et al. 2023; Pedersen et al. 2022). It should be noted that this study does not differentiate between mobile device activities initiated by children or those mediated or initiated by their parents. This limitation is particularly critical for younger age groups, that is, when children are more dependent on their parents to perform such activities and may not use a device yet independently. At this stage, device use is likely heavily influenced by parental decisions and quite likely performed on a parent's device. For instance, our cluster analysis indicates that girls make phone calls more often than boys, with this difference already emerging in the youngest age group of children aged 0–2 years. However, as it is unknown if it is the children or their parents who initiate these activities, it is also unclear if these findings reflect children's behavioral preferences or parental practices.

In line with this observation, our sensitivity analysis indicated that in the NL‐Qualtrics population stricter parental rules may translate to shorter device use, but in older children only. However, this age‐dependent association was not observed in the NL‐CINT or SP‐CINT populations. This may again reflect the greater level of control parents have over the devices used by very young children, rather than an absence of rules controlling device use among these children.

#### 4.6.2 Limitations and Future Directions

To the best of our knowledge, our study is among the first to present detailed frequency of mobile‐device related activities among young children, including even children below 2 years of age. With mobile technology becoming ubiquitous and with its usage starting even before children can talk, this data is of relevance to accurately characterize children's exposure to radiofrequency electromagnetic fields, or to screentime. Accuracy of these calculations is of importance to enable evaluation of possible effects on child health.

As the main limitation of this study, it should be noted that the NL‐CINT and SP‐CINT data sets report overall higher levels of activities than the NL‐QUALT data set, both in number of activities, and, even more clearly in terms of their duration. This is likely due to the different data collection strategies employed for these samples. While the NL‐QUALT data set was collected with a social media campaign on Facebook, the CINT data sets were collected by the company CINT among their panel of participants. Thus, the samples are not very alike, since, unlike the CINT data sets, the NL‐QUALT sample was composed by an overwhelming majority of parents with university education living in non‐urban areas, Moreover, the format of the questionnaires was different in the two data collections: the NL‐QUALT questionnaire asked participants the same set of questions only once for each activity; while in the NL‐CINT questionnaire, the same set of questions for each activity were repeated multiple times, that is, once per each device. This longer and more repetitive format might have brought NL‐CINT respondents to overreporting, or duplicate reporting, compared to the NL‐QUALT respondents. Alternatively, this might be due to an actual difference of usage, maybe due to the different profiles of the samples. Overall, despite the significant differences between these data sets, we report all collected results in order to provide a transparent picture of the work conducted and to provide a range of estimates that future studies can refer to for comparison. In other words, this study illustrates the uncertainty surrounding the amount of time children dedicate to these activities. As it is unclear which data set better reflects the true average frequency and duration of these activities, the results may instead be interpreted as providing plausible ranges of mobile device use frequency and activity durations. For instance, QUAL‐NL reports an average of 7 min of phone calling among 2 year olds, whereas CINT‐NL reports 60 min for the same age group. These findings suggest the true average may lie within this range.

In line with this, while some trends do not replicate across the data sets from the two countries (e.g., gender patterns in video calling, associations with parental age, and gaming trends), it is impossible to tell if this is due to cultural differences between Spain and the Netherlands or rather to differences in the surveys and sampling techniques. Therefore, generalizations regarding country differences should be avoided. Moreover, because neither sample is representative of the respective country's population, the findings cannot be generalized to the populations of Spain or the Netherlands.

Nonetheless, this data can inform scientists aiming to assess exposure to RF‐EMF in children of different ages. The results underline the need to consider not only the duration of use, but also the diversity of activities and the role of parental mediation, that is, parents affecting if and how much children perform activities on mobile devices, when assessing children's exposure to RF‐EMF and a possible consequence for their health. Further studies should explore how the usage patterns at different ages vary also depending on the availability of various devices (smartphones, tablets, laptops…). Future research should also further investigate how variations in usage patterns and parental practices contribute to differences in exposure across age groups and socio‐demographic contexts. For instance, our study shows that the sex differences in device usage exist already before children are 3 years old; this should be considered when estimating young children's exposure levels.

## 5 Conclusion

Overall, this study presents detailed information regarding usage of mobile devices in the Netherlands and in Spain among children from 0 to 11 years of age. We found that in general the number of activities increased with age and that even the very young children are exposed to mobile communication devices. No major consistent patterns were observed in relation to sociodemographic variables apart from older girls generally tending to call more than boys of their ages and older boys gaming more than their feminine counterparts, though reported average duration of use per week did not appear to differ much among users.

Future studies should further explore how usage characteristics (and the related RF‐EMF exposure are associated to different socio‐demographic characteristics of the children and of their families, such as number of children, housing conditions and so forth. If, as some trends in our results suggest, sociodemographic variables significantly affect mobile activities of children, these should be considered when estimating child exposure. Detailed information about the different activities children perform on mobile devices can also inform future studies and policy making on long term effects of mobile devices exposure and on psychological and mental health (see Teague et al. 2026 for a recent meta‐analysis).

## Conflicts of Interest

The authors declare no conflicts of interes.

## Supporting information


Supporting File

## Data Availability

The Dutch and Spanish data sets generated and analysed during the current study may be made available upon request to Anke Huss (a.huss@uu.nl) and Mònica Guxens (monica.guxens@isglobal.org), respectively, in accordance with the local, national, and European Union regulations. The data dictionary and code to reproduce the analysis will be made available upon publication at the open repository ‘dataverse’ of the Consorci de Serveis Universitaris de Catalunya (CSUC).
