# Supplementary material for: Mobile Device Use in Children Aged 0–11 Years—Surveys in the Netherlands and Spain
Source: Bioelectromagnetics. 2026 Oct 21;47(7):e70072. doi: 10.1002/bem.70072 (PMC13686560; doi:10.1002/bem.70072)
Supplement: Supplementary file 1 [file BEM-47-0-s001.docx]

**Supplementary material**

*SM1. Questionnaire samples.*

NL-QUALT questionnaire sample (originally in Dutch).

Does your child use a mobile communication device at least once a week to *make or receive calls (using a cellular network)*? ^[1]^

⬜No

⬜Yes

On average, how long does your child spend *on the phone* with a cell phone or smartphone, and how often per week?^1^

____ minutes per week

____ (number of) times per week

CINT questionnaire sample (originally in Dutch and Spanish for participants).

2. Does your child regularly use a mobile device to *make or receive calls (WITHOUT VIDEO) using an App (e.g. WhatsApp, Hangout, Viber, Skype)*?^1^

⬜ No

⬜ Yes, if so: which device does your child use when *making calls via an app without video*?^1^

⬜ smartphone

⬜ tablet

⬜ laptop connected with WiFi

On average, how much time does your child spend *talking (without video) using an App* on a smartphone?^1,^^[2]^

_______ hours and/or	______minutes	⬜ per day	⬜ per week

*SM2. Stratified tables*

Table A1. NL-QUALT’ dataset summarized for child’s age and child’s sex

|  | **Sex Boy**, N = 388 | | | | | | **Sex Girl**, N = 350 | | | | |
| --- | --- | --- | --- | --- | --- | --- | --- | --- | --- | --- | --- |
| **Variables** | **Overall** N = 388*^1^* | **Age** **0-2**, n = 109*^1^* | **Age** **3-5**, n = 112*^1^* | **Age** **6-8**, n = 99*^1^* | **Age** **9-11**, n = 68*^1^* | **Overall** N = 350*^1^* | | **Age** **0-2**, n = 104*^1^* | **Age** **3-5**, n = 112*^1^* | **Age** **6-8**, n = 76*^1^* | **Age** **9-11**, n = 58*^1^* |
| **Phone calling?** | 123 (32%) | 30 (28%) | 37 (33%) | 24 (24%) | 32 (47%) | 125 (36%) | | 34 (33%) | 34 (30%) | 23 (30%) | 34 (59%) |
| (Missing) | 0 | 0 | 0 | 0 | 0 | 0 | | 0 | 0 | 0 | 0 |
| *minutes per week* | 10 (5, 10) | 6 (5, 15) | 9 (5, 15) | 9 (3, 10) | 10 (5, 10) | 10 (5, 10) | | 9 (5, 10) | 10 (5, 10) | 10 (4, 15) | 10 (5, 10) |
| (Missing) | 279 | 83 | 78 | 77 | 41 | 241 | | 76 | 79 | 57 | 29 |
| **App calling?** | 57 (16%) | 6 (5.8%) | 17 (16%) | 10 (11%) | 24 (39%) | 50 (15%) | | 7 (7.1%) | 14 (13%) | 9 (13%) | 20 (38%) |
| (Missing) | 23 | 5 | 5 | 6 | 7 | 21 | | 6 | 5 | 4 | 6 |
| *minutes per week* | 5.0 (3.0, 10.0) | 5.0 (3.0, 5.0) | 4.0 (2.0, 10.0) | 3.0 (2.0, 5.0) | 5.0 (5.0, 15.0) | 5 (5, 10) | | 10 (5, 10) | 5 (2, 10) | 10 (4, 10) | 5 (5, 15) |
| (Missing) | 342 | 103 | 102 | 90 | 47 | 311 | | 99 | 101 | 70 | 41 |
| **Video calling?** | 104 (30%) | 22 (22%) | 43 (42%) | 14 (15%) | 25 (44%) | 104 (33%) | | 29 (31%) | 42 (41%) | 13 (19%) | 20 (43%) |
| (Missing) | 37 | 8 | 10 | 8 | 11 | 39 | | 10 | 10 | 8 | 11 |
| *minutes per week* | 10 (5, 15) | 10 (4, 15) | 8 (4, 10) | 10 (5, 10) | 10 (5, 20) | 10 (5, 20) | | 15 (5, 30) | 10 (5, 10) | 10 (5, 20) | 10 (5, 30) |
| (Missing) | 293 | 90 | 72 | 86 | 45 | 248 | | 77 | 71 | 60 | 40 |
| **Video streaming?** | 222 (65%) | 31 (32%) | 75 (77%) | 68 (76%) | 48 (86%) | 181 (60%) | | 30 (33%) | 64 (65%) | 52 (74%) | 35 (81%) |
| (Missing) | 46 | 11 | 14 | 9 | 12 | 48 | | 13 | 14 | 6 | 15 |
| *minutes per week* | 120 (35, 300) | 45 (20, 180) | 73 (25, 210) | 150 (60, 420) | 210 (120, 420) | 90 (30, 300) | | 30 (10, 60) | 70 (40, 180) | 210 (60, 350) | 210 (60, 420) |
| (Missing) | 175 | 80 | 38 | 35 | 22 | 176 | | 77 | 49 | 25 | 25 |
| **Audio streaming?** | 116 (35%) | 21 (22%) | 27 (28%) | 34 (40%) | 34 (61%) | 92 (31%) | | 21 (24%) | 25 (26%) | 26 (38%) | 20 (49%) |
| (Missing) | 54 | 12 | 16 | 14 | 12 | 55 | | 17 | 14 | 7 | 17 |
| *minutes per week* | 60 (30, 90) | 30 (15, 60) | 60 (30, 60) | 60 (30, 60) | 90 (40, 120) | 60 (20, 75) | | 20 (10, 60) | 60 (30, 120) | 60 (30, 60) | 60 (20, 120) |
| (Missing) | 273 | 88 | 85 | 64 | 36 | 264 | | 85 | 89 | 51 | 39 |
| **Gaming?** | 119 (36%) | 0 (0%) | 22 (23%) | 49 (58%) | 48 (94%) | 94 (32%) | | 2 (2.4%) | 20 (20%) | 40 (58%) | 32 (82%) |
| (Missing) | 61 | 14 | 16 | 14 | 17 | 59 | | 19 | 14 | 7 | 19 |
| *minutes per week* | 100 (30, 230) | NA (NA, NA) | 30 (20, 60) | 100 (60, 210) | 200 (100, 300) | 60 (30, 150) | | 35 (10, 60) | 60 (30, 90) | 60 (30, 110) | 120 (60, 300) |
| (Missing) | 275 | 109 | 90 | 53 | 23 | 260 | | 102 | 95 | 36 | 27 |
| **Cordless calling?** | 6 (1.9%) | 0 (0%) | 0 (0%) | 0 (0%) | 6 (12%) | 7 (2.5%) | | 1 (1.2%) | 1 (1.1%) | 4 (5.9%) | 1 (2.7%) |
| (Missing) | 67 | 16 | 17 | 16 | 18 | 65 | | 19 | 17 | 8 | 21 |
| *minutes per week* | 0.0 (0.0, 5.0) | 0.0 (0.0, 0.0) | 0.0 (0.0, 0.0) | NA (NA, NA) | 5.0 (0.0, 5.0) | 2.5 (1.0, 5.0) | | 2.5 (2.5, 2.5) | 20.0 (20.0, 20.0) | 1.5 (0.5, 2.5) | 5.0 (5.0, 5.0) |
| (Missing) | 379 | 107 | 111 | 99 | 62 | 343 | | 103 | 111 | 72 | 57 |
| *^1^*n (%); Median (Q1, Q3) | | | | | | | | | | | |

|  | **Sex Boy**, N = 349 | | | | | | **Sex Girl**, N = 319 | | | | |
| --- | --- | --- | --- | --- | --- | --- | --- | --- | --- | --- | --- |
| **Variables** | **Overall** N = 349*^1^* | **Age** **0-2**, n = 58*^1^* | **Age** **3-5**, n = 91*^1^* | **Age** **6-8**, n = 99*^1^* | **Age** **9-11**, n = 101*^1^* | **Overall** N = 319*^1^* | | **Age** **0-2**, n = 52*^1^* | **Age** **3-5**, n = 81*^1^* | **Age** **6-8**, n = 74*^1^* | **Age** **9-11**, n = 112*^1^* |
| **Phone calling?** | 204 (58%) | 20 (34%) | 49 (54%) | 57 (58%) | 78 (77%) | 199 (62%) | | 24 (46%) | 37 (46%) | 45 (61%) | 93 (83%) |
| (Missing) | 0 | 0 | 0 | 0 | 0 | 0 | | 0 | 0 | 0 | 0 |
| *minutes per week* | 120 (50, 420) | 60 (20, 120) | 120 (30, 420) | 105 (50, 240) | 120 (60, 420) | 105 (35, 420) | | 90 (20, 525) | 60 (35, 121) | 98 (48, 420) | 120 (60, 420) |
| (Missing) | 147 | 39 | 42 | 42 | 24 | 121 | | 28 | 44 | 30 | 19 |
| **App calling?** | 175 (51%) | 19 (33%) | 35 (38%) | 58 (59%) | 63 (66%) | 160 (51%) | | 19 (37%) | 29 (36%) | 38 (52%) | 74 (67%) |
| (Missing) | 7 | 1 | 0 | 1 | 5 | 3 | | 0 | 0 | 1 | 2 |
| *minutes per week* | 90 (35, 240) | 70 (30, 105) | 70 (30, 420) | 105 (35, 240) | 105 (60, 210) | 105 (60, 368) | | 210 (60, 840) | 90 (60, 420) | 115 (30, 420) | 105 (60, 210) |
| (Missing) | 174 | 39 | 56 | 41 | 38 | 159 | | 33 | 52 | 36 | 38 |
| **Video calling?** | 160 (47%) | 23 (40%) | 38 (42%) | 45 (46%) | 54 (57%) | 179 (57%) | | 27 (52%) | 42 (52%) | 42 (58%) | 68 (62%) |
| (Missing) | 8 | 1 | 0 | 1 | 6 | 4 | | 0 | 0 | 1 | 3 |
| *minutes per week* | 70 (30, 210) | 70 (30, 90) | 60 (30, 120) | 65 (26, 193) | 105 (60, 360) | 70 (30, 210) | | 70 (15, 210) | 35 (15, 80) | 60 (30, 245) | 120 (60, 300) |
| (Missing) | 190 | 35 | 53 | 55 | 47 | 141 | | 25 | 40 | 32 | 44 |
| **Video streaming?** | 211 (62%) | 25 (44%) | 65 (71%) | 64 (66%) | 57 (60%) | 191 (61%) | | 27 (52%) | 56 (71%) | 46 (63%) | 62 (57%) |
| (Missing) | 9 | 1 | 0 | 2 | 6 | 6 | | 0 | 2 | 1 | 3 |
| *minutes per week* | 420 (210, 770) | 420 (210, 840) | 420 (210, 630) | 420 (210, 634) | 420 (300, 840) | 420 (180, 840) | | 420 (135, 840) | 210 (98, 570) | 424 (210, 840) | 420 (240, 1,050) |
| (Missing) | 138 | 33 | 26 | 35 | 44 | 128 | | 25 | 25 | 28 | 50 |
| **Audio streaming?** | 99 (29%) | 15 (26%) | 28 (31%) | 25 (26%) | 31 (33%) | 87 (28%) | | 14 (27%) | 20 (26%) | 21 (29%) | 32 (30%) |
| (Missing) | 10 | 1 | 0 | 3 | 6 | 11 | | 0 | 3 | 2 | 6 |
| *minutes per week* | 240 (105, 630) | 240 (150, 420) | 420 (70, 630) | 105 (60, 630) | 350 (180, 840) | 210 (60, 511) | | 330 (150, 616) | 193 (33, 473) | 120 (60, 420) | 151 (80, 529) |
| (Missing) | 251 | 43 | 64 | 74 | 70 | 232 | | 38 | 61 | 53 | 80 |
| **Gaming?** | 162 (48%) | 10 (18%) | 33 (37%) | 60 (63%) | 59 (62%) | 140 (45%) | | 11 (21%) | 28 (36%) | 44 (61%) | 57 (54%) |
| (Missing) | 12 | 1 | 2 | 3 | 6 | 11 | | 0 | 3 | 2 | 6 |
| *minutes per week* | 420 (180, 600) | 152 (30, 420) | 315 (120, 420) | 420 (180, 480) | 420 (210, 840) | 420 (135, 427) | | 420 (60, 840) | 315 (88, 420) | 420 (138, 450) | 420 (225, 675) |
| (Missing) | 202 | 48 | 58 | 44 | 52 | 188 | | 41 | 53 | 34 | 60 |
| **Cordless calling?** | 42 (13%) | 7 (12%) | 13 (15%) | 11 (11%) | 11 (12%) | 30 (9.9%) | | 4 (7.7%) | 9 (12%) | 8 (12%) | 9 (8.6%) |
| (Missing) | 13 | 1 | 2 | 3 | 7 | 15 | | 0 | 3 | 5 | 7 |
| *minutes per week* | 175 (60, 420) | 121 (60, 420) | 210 (30, 420) | 300 (60, 861) | 122 (30, 420) | 70 (30, 420) | | 462 (225, 567) | 30 (15, 60) | 83 (20, 420) | 121 (120, 280) |
| (Missing) | 307 | 51 | 78 | 88 | 90 | 289 | | 48 | 72 | 66 | 103 |
| *^1^*n (%); Median (Q1, Q3) | | | | | | | | | | | |

Table A2. NL-CINT’s dataset summarized for child’s age and child’s sex

Table A3. SP-CINT’s dataset summarized for child’s age and child’s sex

|  | **Sex Boy**, N = 226 | | | | **Sex Girl**, N = 180 | | | |
| --- | --- | --- | --- | --- | --- | --- | --- | --- |
| **Variables** | **Overall** N = 226*^1^* | **Age** **3–5**, n = 85*^1^* | **Age** **6–8**, n = 64*^1^* | **Age** **9–11**, n = 77*^1^* | **Overall** N = 180*^1^* | **Age** **3–5**, n = 69*^1^* | **Age** **6–8**, n = 54*^1^* | **Age** **9–11**, n = 57*^1^* |
| **Phone calling?** | 107 (47%) | 32 (38%) | 27 (42%) | 48 (62%) | 77 (43%) | 25 (36%) | 28 (52%) | 24 (42%) |
| (Missing) | 0 | 0 | 0 | 0 | 0 | 0 | 0 | 0 |
| *minutes per week* | 105 (14, 210) | 123 (70, 210) | 105 (14, 210) | 140 (0, 210) | 140 (105, 210) | 210 (105, 210) | 105 (7, 210) | 175 (140, 210) |
| (Missing) | 139 | 59 | 37 | 43 | 123 | 50 | 34 | 39 |
| **App calling?** | 93 (41%) | 27 (32%) | 28 (44%) | 38 (49%) | 70 (39%) | 20 (29%) | 20 (37%) | 30 (53%) |
| (Missing) | 0 | 0 | 0 | 0 | 0 | 0 | 0 | 0 |
| *minutes per week* | 210 (70, 210) | 193 (70, 210) | 210 (70, 210) | 210 (140, 217) | 210 (105, 210) | 210 (105, 210) | 182 (140, 210) | 210 (140, 210) |
| (Missing) | 175 | 67 | 48 | 60 | 141 | 55 | 44 | 42 |
| **Video calling?** | 103 (46%) | 42 (49%) | 29 (45%) | 32 (42%) | 71 (39%) | 27 (39%) | 21 (39%) | 23 (40%) |
| (Missing) | 0 | 0 | 0 | 0 | 0 | 0 | 0 | 0 |
| *minutes per week* | 158 (88, 210) | 105 (70, 210) | 140 (105, 210) | 210 (140, 210) | 210 (140, 210) | 175 (70, 210) | 210 (140, 210) | 210 (140, 280) |
| (Missing) | 162 | 58 | 46 | 58 | 135 | 46 | 45 | 44 |
| **Video streaming?** | 143 (63%) | 61 (72%) | 35 (55%) | 47 (61%) | 112 (62%) | 44 (64%) | 35 (65%) | 33 (58%) |
| (Missing) | 0 | 0 | 0 | 0 | 0 | 0 | 0 | 0 |
| *minutes per week* | 210 (175, 210) | 210 (140, 210) | 210 (210, 210) | 210 (140, 315) | 210 (210, 210) | 210 (210, 210) | 210 (210, 210) | 210 (210, 210) |
| (Missing) | 173 | 63 | 48 | 62 | 131 | 49 | 38 | 44 |
| **Audio streaming?** | 57 (25%) | 19 (22%) | 12 (19%) | 26 (34%) | 52 (29%) | 18 (26%) | 15 (28%) | 19 (33%) |
| (Missing) | 0 | 0 | 0 | 0 | 0 | 0 | 0 | 0 |
| *minutes per week* | 210 (105, 210) | 210 (70, 210) | 210 (105, 210) | 193 (112, 210) | 210 (105, 315) | 210 (70, 368) | 210 (210, 315) | 105 (105, 140) |
| (Missing) | 196 | 74 | 57 | 65 | 161 | 61 | 48 | 52 |
| **Gaming?** | 95 (42%) | 27 (32%) | 28 (44%) | 40 (52%) | 77 (43%) | 21 (30%) | 22 (41%) | 34 (60%) |
| (Missing) | 0 | 0 | 0 | 0 | 0 | 0 | 0 | 0 |
| *minutes per week* | 210 (140, 210) | 179 (140, 280) | 210 (158, 210) | 210 (140, 210) | 193 (88, 210) | 210 (175, 210) | 168 (70, 210) | 140 (70, 210) |
| (Missing) | 192 | 75 | 52 | 65 | 152 | 57 | 49 | 46 |
| **Cordless calling?** | 25 (11%) | 9 (11%) | 7 (11%) | 9 (12%) | 24 (13%) | 10 (14%) | 6 (11%) | 8 (14%) |
| (Missing) | 0 | 0 | 0 | 0 | 0 | 0 | 0 | 0 |
| *minutes per week* | 95 (35, 210) | 70 (0, 210) | 210 (70, 315) | 95 (70, 140) | 105 (35, 210) | 70 (35, 210) | 105 (0, 210) | 105 (35, 210) |
| (Missing) | 208 | 78 | 59 | 71 | 162 | 60 | 51 | 51 |
| *^1^*n (%); Median (Q1, Q3) | | | | | | | | |

Table B1. NL-QUALT’s dataset summarized for child’s age and parent’s age

|  | **Parent's age <30**, N = 112 | | | | | **Parent's age 30<40**, N = 565 | | | | | **Parent's age ≥40**, N = 61 | | | | |
| --- | --- | --- | --- | --- | --- | --- | --- | --- | --- | --- | --- | --- | --- | --- | --- |
| **Variables** | **Overall** N = 112*^1^* | **Age** **0-2**, n = 26*^1^* | **Age** **3-5**, n = 32*^1^* | **Age** **6-8**, n = 33*^1^* | **Age** **9-11**, n = 21*^1^* | **Overall** N = 565*^1^* | **Age** **0-2**, n = 174*^1^* | **Age** **3-5**, n = 169*^1^* | **Age** **6-8**, n = 125*^1^* | **Age** **9-11**, n = 97*^1^* | **Overall** N = 61*^1^* | **Age** **0-2**, n = 12*^1^* | **Age** **3-5**, n = 24*^1^* | **Age** **6-8**, n = 16*^1^* | **Age** **9-11**, n = 9*^1^* |
| **Phone calling?** | 37 (33%) | 6 (23%) | 9 (28%) | 13 (39%) | 9 (43%) | 187 (33%) | 53 (30%) | 55 (33%) | 26 (21%) | 53 (55%) | 26 (43%) | 5 (42%) | 8 (33%) | 8 (50%) | 5 (56%) |
| (Missing) | 0 | 0 | 0 | 0 | 0 | 0 | 0 | 0 | 0 | 0 | 0 | 0 | 0 | 0 | 0 |
| *minutes per week* | 5 (4, 10) | 10 (10, 15) | 5 (2, 8) | 5 (3, 10) | 5 (3, 10) | 10 (5, 10) | 5 (5, 15) | 10 (5, 15) | 10 (5, 15) | 10 (5, 10) | 10 (5, 15) | 5 (3, 10) | 9 (7, 30) | 10 (6, 18) | 10 (5, 15) |
| (Missing) | 80 | 20 | 24 | 22 | 14 | 400 | 131 | 116 | 103 | 50 | 39 | 7 | 18 | 8 | 6 |
| **App calling?** | 20 (19%) | 3 (12%) | 3 (10%) | 6 (20%) | 8 (44%) | 78 (15%) | 9 (5.5%) | 27 (17%) | 10 (8.5%) | 32 (36%) | 10 (18%) | 1 (9.1%) | 1 (4.8%) | 3 (19%) | 5 (63%) |
| (Missing) | 8 | 0 | 2 | 3 | 3 | 32 | 10 | 6 | 7 | 9 | 5 | 1 | 3 | 0 | 1 |
| *minutes per week* | 5.00 (4.00, 10.00) | 10.00 (5.00, 10.00) | 6.00 (2.00, 10.00) | 2.00 (2.00, 4.00) | 5.00 (5.00, 10.00) | 5 (3, 10) | 5 (3, 5) | 5 (2, 10) | 5 (2, 10) | 5 (5, 15) | 5.0 (5.0, 12.5) | 5.0 (5.0, 5.0) | 2.0 (2.0, 2.0) | 10.0 (5.0, 15.0) | 5.0 (5.0, 15.0) |
| (Missing) | 95 | 23 | 30 | 28 | 14 | 504 | 167 | 151 | 118 | 68 | 53 | 11 | 23 | 13 | 6 |
| **Video calling?** | 33 (34%) | 6 (24%) | 13 (45%) | 6 (21%) | 8 (50%) | 162 (32%) | 41 (26%) | 68 (44%) | 17 (15%) | 36 (43%) | 14 (27%) | 4 (36%) | 4 (20%) | 4 (25%) | 2 (40%) |
| (Missing) | 14 | 1 | 3 | 5 | 5 | 54 | 16 | 14 | 11 | 13 | 9 | 1 | 4 | 0 | 4 |
| *minutes per week* | 10 (5, 15) | 30 (10, 30) | 10 (5, 10) | 10 (5, 15) | 10 (5, 20) | 10 (5, 15) | 10 (5, 20) | 8 (5, 10) | 10 (5, 15) | 10 (5, 30) | 10 (5, 20) | 8 (4, 15) | 6 (4, 19) | 15 (10, 23) | 13 (5, 20) |
| (Missing) | 80 | 21 | 19 | 26 | 14 | 413 | 137 | 105 | 107 | 64 | 47 | 8 | 20 | 12 | 7 |
| **Video streaming?** | 50 (52%) | 6 (25%) | 15 (54%) | 19 (63%) | 10 (71%) | 313 (63%) | 49 (32%) | 109 (73%) | 87 (76%) | 68 (85%) | 39 (76%) | 6 (55%) | 15 (79%) | 13 (87%) | 5 (83%) |
| (Missing) | 16 | 2 | 4 | 3 | 7 | 69 | 21 | 20 | 11 | 17 | 10 | 1 | 5 | 1 | 3 |
| *minutes per week* | 113 (60, 220) | 30 (20, 70) | 60 (40, 180) | 150 (60, 500) | 200 (100, 420) | 120 (30, 300) | 30 (15, 120) | 73 (30, 200) | 210 (60, 375) | 210 (85, 420) | 105 (30, 360) | 48 (10, 70) | 75 (30, 300) | 180 (120, 420) | 30 (30, 600) |
| (Missing) | 62 | 20 | 17 | 14 | 11 | 266 | 130 | 61 | 42 | 33 | 23 | 6 | 10 | 3 | 4 |
| **Audio streaming?** | 31 (32%) | 5 (21%) | 8 (29%) | 10 (33%) | 8 (57%) | 164 (34%) | 34 (23%) | 42 (28%) | 44 (41%) | 44 (56%) | 13 (25%) | 3 (27%) | 2 (11%) | 6 (38%) | 2 (33%) |
| (Missing) | 16 | 2 | 4 | 3 | 7 | 83 | 26 | 21 | 17 | 19 | 10 | 1 | 6 | 0 | 3 |
| *minutes per week* | 60 (30, 120) | 120 (25, 255) | 60 (45, 150) | 48 (30, 80) | 60 (40, 120) | 60 (30, 90) | 20 (10, 60) | 60 (30, 100) | 60 (30, 60) | 65 (30, 120) | 60 (30, 60) | 30 (15, 60) | 60 (60, 60) | 60 (30, 60) | 85 (20, 150) |
| (Missing) | 83 | 22 | 24 | 23 | 14 | 406 | 141 | 129 | 81 | 55 | 48 | 9 | 22 | 10 | 7 |
| **Gaming?** | 27 (30%) | 0 (0%) | 2 (7.4%) | 12 (40%) | 13 (100%) | 167 (35%) | 2 (1.4%) | 38 (26%) | 66 (61%) | 61 (85%) | 19 (38%) | 0 (0%) | 2 (11%) | 11 (73%) | 6 (100%) |
| (Missing) | 21 | 5 | 5 | 3 | 8 | 88 | 27 | 20 | 16 | 25 | 11 | 1 | 6 | 1 | 3 |
| *minutes per week* | 110 (30, 230) | NA (NA, NA) | 60 (30, 90) | 135 (45, 210) | 120 (60, 300) | 65 (30, 200) | 35 (10, 60) | 30 (20, 60) | 60 (30, 150) | 150 (100, 300) | 150 (60, 300) | NA (NA, NA) | 18 (15, 20) | 95 (60, 390) | 225 (210, 300) |
| (Missing) | 85 | 26 | 30 | 21 | 8 | 409 | 172 | 134 | 63 | 40 | 41 | 12 | 22 | 4 | 3 |
| **Cordless calling?** | 1 (1.1%) | 0 (0%) | 0 (0%) | 1 (3.3%) | 0 (0%) | 11 (2.4%) | 1 (0.7%) | 1 (0.7%) | 3 (2.9%) | 6 (8.7%) | 1 (2.0%) | 0 (0%) | 0 (0%) | 0 (0%) | 1 (17%) |
| (Missing) | 22 | 5 | 6 | 3 | 8 | 99 | 28 | 23 | 20 | 28 | 11 | 2 | 6 | 0 | 3 |
| *minutes per week* | 1.0000 (1.0000, 1.0000) | NA (NA, NA) | NA (NA, NA) | 1.0000 (1.0000, 1.0000) | NA (NA, NA) | 3.0 (0.0, 5.0) | 1.3 (0.0, 2.5) | 10.0 (0.0, 20.0) | 2.0 (0.0, 3.0) | 5.0 (5.0, 5.0) | 0.0000 (0.0000, 0.0000) | 0.0000 (0.0000, 0.0000) | NA (NA, NA) | NA (NA, NA) | 0.0000 (0.0000, 0.0000) |
| (Missing) | 111 | 26 | 32 | 32 | 21 | 552 | 172 | 167 | 122 | 91 | 59 | 11 | 24 | 16 | 8 |
| *^1^*n (%); Median (Q1, Q3) | | | | | | | | | | | |  |  |  |  |

Table B2. NL-CINT’s dataset summarized for child’s age and parent’s age

|  | **Parent's age <30**, N = 362 | | | | | **Parent's age 30<40**, N = 259 | | | | | **Parent's age ≥40**, N = 47 | | | | |
| --- | --- | --- | --- | --- | --- | --- | --- | --- | --- | --- | --- | --- | --- | --- | --- |
| **Variables** | **Overall** N = 362*^1^* | **Age** **0-2**, n = 46*^1^* | **Age** **3-5**, n = 94*^1^* | **Age** **6-8**, n = 98*^1^* | **Age** **9-11**, n = 124*^1^* | **Overall** N = 259*^1^* | **Age** **0-2**, n = 52*^1^* | **Age** **3-5**, n = 69*^1^* | **Age** **6-8**, n = 64*^1^* | **Age** **9-11**, n = 74*^1^* | **Overall** N = 47*^1^* | **Age** **0-2**, n = 12*^1^* | **Age** **3-5**, n = 9*^1^* | **Age** **6-8**, n = 11*^1^* | **Age** **9-11**, n = 15*^1^* |
| **Phone calling?** | 235 (65%) | 21 (46%) | 48 (51%) | 65 (66%) | 101 (81%) | 140 (54%) | 13 (25%) | 36 (52%) | 34 (53%) | 57 (77%) | 28 (60%) | 10 (83%) | 2 (22%) | 3 (27%) | 13 (87%) |
| (Missing) | 0 | 0 | 0 | 0 | 0 | 0 | 0 | 0 | 0 | 0 | 0 | 0 | 0 | 0 | 0 |
| *minutes per week* | 120 (45, 420) | 35 (10, 120) | 70 (35, 315) | 120 (60, 420) | 120 (60, 420) | 110 (35, 420) | 420 (30, 861) | 75 (30, 165) | 60 (30, 180) | 140 (60, 420) | 120 (60, 240) | 120 (35, 120) | 88 (35, 140) | 105 (65, 210) | 210 (63, 420) |
| (Missing) | 128 | 25 | 46 | 33 | 24 | 120 | 39 | 33 | 31 | 17 | 20 | 3 | 7 | 8 | 2 |
| **App calling?** | 202 (57%) | 15 (33%) | 42 (45%) | 64 (66%) | 81 (68%) | 112 (44%) | 16 (31%) | 18 (26%) | 29 (46%) | 49 (67%) | 21 (47%) | 7 (64%) | 4 (44%) | 3 (27%) | 7 (50%) |
| (Missing) | 6 | 0 | 0 | 1 | 5 | 2 | 0 | 0 | 1 | 1 | 2 | 1 | 0 | 0 | 1 |
| *minutes per week* | 105 (40, 300) | 60 (30, 140) | 90 (40, 420) | 140 (39, 420) | 90 (45, 210) | 90 (35, 210) | 158 (35, 665) | 80 (30, 315) | 60 (30, 133) | 105 (60, 210) | 105 (60, 420) | 105 (35, 420) | 43 (13, 280) | 140 (60, 1,295) | 180 (60, 420) |
| (Missing) | 160 | 31 | 52 | 34 | 43 | 147 | 36 | 51 | 35 | 25 | 26 | 5 | 5 | 8 | 8 |
| **Video calling?** | 190 (54%) | 22 (48%) | 45 (48%) | 51 (53%) | 72 (61%) | 125 (49%) | 18 (35%) | 32 (46%) | 31 (49%) | 44 (60%) | 24 (55%) | 10 (91%) | 3 (33%) | 5 (45%) | 6 (46%) |
| (Missing) | 7 | 0 | 0 | 1 | 6 | 2 | 0 | 0 | 1 | 1 | 3 | 1 | 0 | 0 | 2 |
| *minutes per week* | 70 (30, 240) | 60 (20, 120) | 60 (15, 70) | 70 (21, 420) | 105 (60, 325) | 60 (30, 210) | 70 (30, 150) | 60 (23, 145) | 55 (30, 155) | 120 (48, 320) | 70 (30, 105) | 70 (35, 90) | 30 (5, 35) | 70 (60, 70) | 330 (30, 420) |
| (Missing) | 173 | 24 | 50 | 47 | 52 | 135 | 34 | 37 | 34 | 30 | 23 | 2 | 6 | 6 | 9 |
| **Video streaming?** | 217 (61%) | 21 (46%) | 66 (71%) | 60 (62%) | 70 (59%) | 157 (62%) | 25 (48%) | 50 (74%) | 40 (65%) | 42 (58%) | 28 (64%) | 6 (55%) | 5 (56%) | 10 (91%) | 7 (54%) |
| (Missing) | 8 | 0 | 1 | 1 | 6 | 4 | 0 | 1 | 2 | 1 | 3 | 1 | 0 | 0 | 2 |
| *minutes per week* | 420 (180, 840) | 420 (210, 840) | 330 (120, 630) | 420 (210, 840) | 420 (240, 870) | 420 (210, 630) | 420 (140, 770) | 420 (210, 630) | 420 (210, 615) | 420 (300, 840) | 420 (225, 630) | 420 (313, 630) | 315 (210, 420) | 630 (420, 1,050) | 240 (210, 480) |
| (Missing) | 145 | 25 | 28 | 38 | 54 | 102 | 27 | 19 | 24 | 32 | 19 | 6 | 4 | 1 | 8 |
| **Audio streaming?** | 106 (30%) | 12 (26%) | 29 (32%) | 26 (27%) | 39 (33%) | 64 (25%) | 9 (17%) | 16 (24%) | 16 (26%) | 23 (32%) | 16 (36%) | 8 (73%) | 3 (33%) | 4 (36%) | 1 (7.7%) |
| (Missing) | 11 | 0 | 2 | 2 | 7 | 7 | 0 | 1 | 3 | 3 | 3 | 1 | 0 | 0 | 2 |
| *minutes per week* | 270 (90, 630) | 368 (135, 728) | 420 (90, 630) | 203 (60, 840) | 240 (120, 630) | 210 (60, 420) | 420 (150, 420) | 210 (30, 420) | 83 (60, 420) | 240 (105, 525) | 225 (113, 525) | 225 (210, 330) | 420 (20, 630) | 83 (60, 473) | 1,155 (1,155, 1,155) |
| (Missing) | 256 | 34 | 65 | 72 | 85 | 196 | 43 | 54 | 48 | 51 | 31 | 4 | 6 | 7 | 14 |
| **Gaming?** | 168 (48%) | 7 (15%) | 33 (36%) | 59 (61%) | 69 (59%) | 112 (45%) | 9 (17%) | 27 (40%) | 36 (59%) | 40 (56%) | 22 (50%) | 5 (45%) | 1 (11%) | 9 (82%) | 7 (54%) |
| (Missing) | 12 | 0 | 3 | 2 | 7 | 8 | 0 | 2 | 3 | 3 | 3 | 1 | 0 | 0 | 2 |
| *minutes per week* | 420 (180, 601) | 427 (30, 560) | 315 (120, 420) | 420 (160, 540) | 420 (210, 840) | 341 (140, 420) | 210 (60, 420) | 240 (70, 420) | 210 (120, 420) | 420 (300, 840) | 315 (180, 420) | 180 (35, 420) | 315 (315, 315) | 420 (420, 840) | 150 (30, 240) |
| (Missing) | 209 | 39 | 61 | 46 | 63 | 155 | 43 | 42 | 30 | 40 | 26 | 7 | 8 | 2 | 9 |
| **Cordless calling?** | 45 (13%) | 6 (13%) | 11 (12%) | 16 (17%) | 12 (10%) | 22 (8.8%) | 2 (3.8%) | 10 (15%) | 2 (3.3%) | 8 (11%) | 5 (11%) | 3 (27%) | 1 (11%) | 1 (9.1%) | 0 (0%) |
| (Missing) | 16 | 0 | 3 | 5 | 8 | 9 | 0 | 2 | 3 | 4 | 3 | 1 | 0 | 0 | 2 |
| *minutes per week* | 121 (60, 420) | 462 (60, 630) | 60 (15, 80) | 360 (53, 438) | 122 (90, 245) | 95 (30, 420) | 245 (70, 420) | 135 (30, 360) | 65 (10, 120) | 135 (18, 420) | 30 (20, 121) | 121 (30, 420) | 15 (15, 15) | 20 (20, 20) | NA (NA, NA) |
| (Missing) | 317 | 40 | 83 | 82 | 112 | 237 | 50 | 59 | 62 | 66 | 42 | 9 | 8 | 10 | 15 |
| *^1^*n (%); Median (Q1, Q3) | | | | | | | | | | | |  |  |  |  |

Table B3. SP-CINT’s dataset summarized for child’s age and parent’s age

|  | **Parent's age <30**, N = 156 | | | | **Parent's age 30<40**, N = 202 | | | | **Parent's age ≥40**, N = 48 | | | |
| --- | --- | --- | --- | --- | --- | --- | --- | --- | --- | --- | --- | --- |
| **Variables** | **Overall** N = 156*^1^* | **Age** **3–5**, n = 70*^1^* | **Age** **6–8**, n = 35*^1^* | **Age** **9–11**, n = 51*^1^* | **Overall** N = 202*^1^* | **Age** **3–5**, n = 68*^1^* | **Age** **6–8**, n = 63*^1^* | **Age** **9–11**, n = 71*^1^* | **Overall** N = 48*^1^* | **Age** **3–5**, n = 16*^1^* | **Age** **6–8**, n = 20*^1^* | **Age** **9–11**, n = 12*^1^* |
| **Phone calling?** | 79 (51%) | 27 (39%) | 21 (60%) | 31 (61%) | 84 (42%) | 24 (35%) | 25 (40%) | 35 (49%) | 21 (44%) | 6 (38%) | 9 (45%) | 6 (50%) |
| (Missing) | 0 | 0 | 0 | 0 | 0 | 0 | 0 | 0 | 0 | 0 | 0 | 0 |
| *minutes per week* | 140 (53, 210) | 210 (105, 210) | 105 (14, 210) | 140 (35, 210) | 140 (28, 210) | 210 (70, 210) | 70 (0, 210) | 140 (49, 210) | 105 (0, 210) | 105 (70, 105) | 158 (105, 210) | 0 (0, 105) |
| (Missing) | 100 | 54 | 18 | 28 | 133 | 45 | 41 | 47 | 29 | 10 | 12 | 7 |
| **App calling?** | 69 (44%) | 23 (33%) | 17 (49%) | 29 (57%) | 77 (38%) | 18 (26%) | 27 (43%) | 32 (45%) | 17 (35%) | 6 (38%) | 4 (20%) | 7 (58%) |
| (Missing) | 0 | 0 | 0 | 0 | 0 | 0 | 0 | 0 | 0 | 0 | 0 | 0 |
| *minutes per week* | 210 (105, 210) | 210 (105, 210) | 140 (70, 210) | 210 (123, 249) | 210 (140, 210) | 210 (140, 210) | 210 (70, 210) | 210 (140, 210) | 105 (70, 210) | 70 (70, 105) | 210 (210, 210) | 70 (70, 70) |
| (Missing) | 120 | 59 | 26 | 35 | 157 | 52 | 49 | 56 | 39 | 11 | 17 | 11 |
| **Video calling?** | 71 (46%) | 32 (46%) | 17 (49%) | 22 (43%) | 84 (42%) | 29 (43%) | 28 (44%) | 27 (38%) | 19 (40%) | 8 (50%) | 5 (25%) | 6 (50%) |
| (Missing) | 0 | 0 | 0 | 0 | 0 | 0 | 0 | 0 | 0 | 0 | 0 | 0 |
| *minutes per week* | 140 (70, 210) | 175 (70, 210) | 175 (140, 210) | 140 (140, 210) | 210 (105, 210) | 158 (105, 210) | 210 (105, 210) | 210 (210, 210) | 105 (105, 210) | 105 (70, 210) | 123 (70, 175) | 175 (140, 210) |
| (Missing) | 113 | 49 | 27 | 37 | 149 | 46 | 48 | 55 | 35 | 9 | 16 | 10 |
| **Video streaming?** | 112 (72%) | 55 (79%) | 23 (66%) | 34 (67%) | 119 (59%) | 42 (62%) | 37 (59%) | 40 (56%) | 24 (50%) | 8 (50%) | 10 (50%) | 6 (50%) |
| (Missing) | 0 | 0 | 0 | 0 | 0 | 0 | 0 | 0 | 0 | 0 | 0 | 0 |
| *minutes per week* | 210 (210, 217) | 210 (140, 210) | 210 (210, 385) | 210 (210, 210) | 210 (210, 210) | 210 (210, 298) | 210 (140, 210) | 210 (175, 263) | 175 (140, 210) | 140 (140, 210) | 210 (210, 280) | 140 (105, 175) |
| (Missing) | 117 | 51 | 24 | 42 | 148 | 48 | 45 | 55 | 39 | 13 | 17 | 9 |
| **Audio streaming?** | 48 (31%) | 20 (29%) | 9 (26%) | 19 (37%) | 51 (25%) | 13 (19%) | 16 (25%) | 22 (31%) | 10 (21%) | 4 (25%) | 2 (10%) | 4 (33%) |
| (Missing) | 0 | 0 | 0 | 0 | 0 | 0 | 0 | 0 | 0 | 0 | 0 | 0 |
| *minutes per week* | 175 (70, 210) | 88 (70, 280) | 210 (210, 420) | 140 (70, 210) | 210 (105, 210) | 210 (210, 315) | 210 (210, 210) | 140 (105, 210) | 210 (70, 210) | 140 (70, 210) | NA (NA, NA) | 210 (210, 210) |
| (Missing) | 138 | 62 | 32 | 44 | 174 | 59 | 53 | 62 | 45 | 14 | 20 | 11 |
| **Gaming?** | 66 (42%) | 27 (39%) | 15 (43%) | 24 (47%) | 88 (44%) | 19 (28%) | 28 (44%) | 41 (58%) | 18 (38%) | 2 (13%) | 7 (35%) | 9 (75%) |
| (Missing) | 0 | 0 | 0 | 0 | 0 | 0 | 0 | 0 | 0 | 0 | 0 | 0 |
| *minutes per week* | 210 (70, 210) | 210 (147, 210) | 88 (21, 280) | 140 (70, 210) | 210 (140, 210) | 175 (140, 280) | 210 (210, 210) | 175 (105, 210) | 210 (210, 210) | 210 (210, 210) | 140 (70, 210) | 210 (210, 210) |
| (Missing) | 131 | 59 | 29 | 43 | 170 | 58 | 54 | 58 | 43 | 15 | 18 | 10 |
| **Cordless calling?** | 18 (12%) | 7 (10%) | 4 (11%) | 7 (14%) | 23 (11%) | 9 (13%) | 8 (13%) | 6 (8.5%) | 8 (17%) | 3 (19%) | 1 (5.0%) | 4 (33%) |
| (Missing) | 0 | 0 | 0 | 0 | 0 | 0 | 0 | 0 | 0 | 0 | 0 | 0 |
| *minutes per week* | 77 (35, 140) | 88 (35, 210) | 105 (0, 385) | 70 (35, 84) | 70 (21, 210) | 46 (0, 123) | 210 (70, 210) | 105 (56, 158) | 210 (210, 210) | 263 (210, 315) | NA (NA, NA) | 210 (105, 210) |
| (Missing) | 142 | 64 | 32 | 46 | 185 | 60 | 58 | 67 | 43 | 14 | 20 | 9 |
| *^1^*n (%); Median (Q1, Q3) | | | | | | | | | |  |  |  |

Table C1. NL-QUALT’s dataset summarized for child’s age and parent’s education

|  | **Parent's education No-University**, N = 71 | | | | | **Parent's education University**, N = 667 | | | | |
| --- | --- | --- | --- | --- | --- | --- | --- | --- | --- | --- |
| **Variables** | **Overall** N = 71*^1^* | **Age** **0-2**, n = 9*^1^* | **Age** **3-5**, n = 16*^1^* | **Age** **6-8**, n = 26*^1^* | **Age** **9-11**, n = 20*^1^* | **Overall** N = 667*^1^* | **Age** **0-2**, n = 203*^1^* | **Age** **3-5**, n = 209*^1^* | **Age** **6-8**, n = 149*^1^* | **Age** **9-11**, n = 106*^1^* |
| **Phone calling?** | 27 (38%) | 0 (0%) | 4 (25%) | 8 (31%) | 15 (75%) | 222 (33%) | 64 (32%) | 68 (33%) | 39 (26%) | 51 (48%) |
| (Missing) | 0 | 0 | 0 | 0 | 0 | 0 | 0 | 0 | 0 | 0 |
| *minutes per week* | 10.0 (5.0, 10.0) | NA (NA, NA) | 10.0 (7.0, 20.0) | 5.0 (2.0, 10.0) | 10.0 (5.0, 10.0) | 10 (5, 10) | 7 (5, 15) | 9 (5, 13) | 10 (5, 15) | 10 (5, 10) |
| (Missing) | 52 | 9 | 13 | 21 | 9 | 468 | 149 | 145 | 113 | 61 |
| **App calling?** | 19 (30%) | 1 (11%) | 4 (27%) | 3 (14%) | 11 (65%) | 88 (14%) | 12 (6.3%) | 27 (14%) | 16 (11%) | 33 (34%) |
| (Missing) | 8 | 0 | 1 | 4 | 3 | 37 | 11 | 10 | 6 | 10 |
| *minutes per week* | 5.0 (5.0, 10.0) | 10.0 (10.0, 10.0) | 2.0 (2.0, 2.0) | 6.0 (2.0, 10.0) | 5.0 (5.0, 15.0) | 5.0 (3.0, 10.0) | 5.0 (3.0, 5.0) | 5.0 (2.0, 10.0) | 5.0 (2.0, 10.0) | 7.5 (5.0, 15.0) |
| (Missing) | 58 | 8 | 15 | 24 | 11 | 594 | 193 | 189 | 136 | 76 |
| **Video calling?** | 20 (33%) | 2 (22%) | 4 (29%) | 4 (18%) | 10 (67%) | 189 (31%) | 49 (26%) | 81 (43%) | 23 (17%) | 36 (40%) |
| (Missing) | 11 | 0 | 2 | 4 | 5 | 65 | 18 | 19 | 12 | 16 |
| *minutes per week* | 10 (6, 30) | NA (NA, NA) | 10 (7, 15) | 10 (7, 20) | 20 (5, 30) | 10 (5, 15) | 10 (5, 25) | 9 (5, 10) | 10 (5, 15) | 10 (5, 20) |
| (Missing) | 55 | 9 | 13 | 22 | 11 | 485 | 157 | 131 | 124 | 73 |
| **Video streaming?** | 37 (67%) | 2 (29%) | 8 (62%) | 15 (68%) | 12 (92%) | 365 (62%) | 58 (32%) | 131 (72%) | 105 (76%) | 71 (82%) |
| (Missing) | 16 | 2 | 3 | 4 | 7 | 78 | 22 | 26 | 11 | 19 |
| *minutes per week* | 210 (100, 420) | 50 (30, 70) | 190 (90, 335) | 360 (120, 840) | 200 (180, 420) | 100 (30, 300) | 30 (15, 120) | 60 (30, 180) | 150 (60, 360) | 210 (60, 420) |
| (Missing) | 37 | 7 | 8 | 12 | 10 | 315 | 150 | 80 | 48 | 37 |
| **Audio streaming?** | 19 (36%) | 3 (43%) | 2 (15%) | 7 (33%) | 7 (58%) | 188 (33%) | 38 (22%) | 50 (28%) | 53 (40%) | 47 (55%) |
| (Missing) | 18 | 2 | 3 | 5 | 8 | 91 | 27 | 28 | 16 | 20 |
| *minutes per week* | 60 (35, 120) | 60 (30, 120) | 60 (60, 60) | 60 (50, 180) | 60 (10, 120) | 60 (30, 90) | 25 (10, 60) | 60 (30, 110) | 60 (30, 60) | 60 (40, 120) |
| (Missing) | 53 | 6 | 14 | 19 | 14 | 485 | 167 | 161 | 96 | 61 |
| **Gaming?** | 25 (49%) | 0 (0%) | 2 (15%) | 12 (60%) | 11 (100%) | 188 (33%) | 2 (1.2%) | 40 (22%) | 77 (57%) | 69 (86%) |
| (Missing) | 20 | 2 | 3 | 6 | 9 | 100 | 31 | 28 | 15 | 26 |
| *minutes per week* | 120 (60, 210) | NA (NA, NA) | 90 (60, 120) | 150 (60, 225) | 120 (30, 180) | 70 (30, 210) | 35 (10, 60) | 30 (20, 60) | 60 (40, 150) | 200 (100, 300) |
| (Missing) | 46 | 9 | 14 | 14 | 9 | 489 | 201 | 172 | 75 | 41 |
| **Cordless calling?** | 3 (5.9%) | 1 (14%) | 0 (0%) | 1 (4.8%) | 1 (10%) | 10 (1.8%) | 0 (0%) | 1 (0.6%) | 3 (2.3%) | 6 (7.7%) |
| (Missing) | 20 | 2 | 3 | 5 | 10 | 112 | 33 | 32 | 19 | 28 |
| *minutes per week* | 3.75 (2.50, 5.00) | 2.50 (2.50, 2.50) | NA (NA, NA) | NA (NA, NA) | 5.00 (5.00, 5.00) | 1.5 (0.0, 5.0) | 0.0 (0.0, 0.0) | 10.0 (0.0, 20.0) | 1.5 (0.5, 2.5) | 5.0 (0.0, 5.0) |
| (Missing) | 69 | 8 | 16 | 26 | 19 | 653 | 201 | 207 | 145 | 100 |
| *^1^*n (%); Median (Q1, Q3) | | | | | | | | | | |

Table C2. NL-CINT’s dataset summarized for child’s age and parent’s education

|  | **Parent's education No-University**, N = 324 | | | | | **Parent's education University**, N = 344 | | | | |
| --- | --- | --- | --- | --- | --- | --- | --- | --- | --- | --- |
| **Variables** | **Overall** N = 324*^1^* | **Age** **0-2**, n = 40*^1^* | **Age** **3-5**, n = 88*^1^* | **Age** **6-8**, n = 82*^1^* | **Age** **9-11**, n = 114*^1^* | **Overall** N = 344*^1^* | **Age** **0-2**, n = 70*^1^* | **Age** **3-5**, n = 84*^1^* | **Age** **6-8**, n = 91*^1^* | **Age** **9-11**, n = 99*^1^* |
| **Phone calling?** | 209 (65%) | 19 (48%) | 52 (59%) | 51 (62%) | 87 (76%) | 194 (56%) | 25 (36%) | 34 (40%) | 51 (56%) | 84 (85%) |
| (Missing) | 0 | 0 | 0 | 0 | 0 | 0 | 0 | 0 | 0 | 0 |
| *minutes per week* | 120 (60, 420) | 120 (30, 840) | 105 (35, 315) | 105 (60, 210) | 120 (60, 420) | 105 (35, 420) | 35 (10, 125) | 60 (30, 150) | 90 (30, 420) | 130 (60, 424) |
| (Missing) | 117 | 21 | 36 | 32 | 28 | 151 | 46 | 50 | 40 | 15 |
| **App calling?** | 171 (54%) | 20 (50%) | 36 (41%) | 45 (56%) | 70 (65%) | 164 (48%) | 18 (26%) | 28 (33%) | 51 (57%) | 67 (68%) |
| (Missing) | 7 | 0 | 0 | 1 | 6 | 3 | 1 | 0 | 1 | 1 |
| *minutes per week* | 105 (40, 284) | 105 (65, 420) | 98 (33, 420) | 133 (60, 420) | 70 (35, 210) | 98 (35, 240) | 48 (20, 210) | 70 (33, 368) | 75 (30, 240) | 105 (60, 300) |
| (Missing) | 153 | 20 | 52 | 37 | 44 | 180 | 52 | 56 | 40 | 32 |
| **Video calling?** | 177 (56%) | 25 (63%) | 42 (48%) | 47 (58%) | 63 (59%) | 162 (48%) | 25 (36%) | 38 (45%) | 40 (44%) | 59 (61%) |
| (Missing) | 8 | 0 | 0 | 1 | 7 | 4 | 1 | 0 | 1 | 2 |
| *minutes per week* | 70 (30, 210) | 70 (35, 150) | 60 (20, 105) | 65 (30, 155) | 105 (60, 280) | 70 (30, 245) | 60 (15, 120) | 55 (15, 70) | 60 (30, 368) | 120 (60, 360) |
| (Missing) | 149 | 15 | 47 | 36 | 51 | 182 | 45 | 46 | 51 | 40 |
| **Video streaming?** | 192 (61%) | 18 (45%) | 62 (71%) | 53 (66%) | 59 (55%) | 210 (62%) | 34 (49%) | 59 (71%) | 57 (63%) | 60 (62%) |
| (Missing) | 10 | 0 | 1 | 2 | 7 | 5 | 1 | 1 | 1 | 2 |
| *minutes per week* | 420 (210, 840) | 420 (420, 1,260) | 420 (140, 630) | 420 (210, 840) | 630 (240, 840) | 420 (210, 630) | 368 (135, 630) | 315 (150, 600) | 420 (210, 630) | 420 (300, 847) |
| (Missing) | 132 | 22 | 26 | 29 | 55 | 134 | 36 | 25 | 34 | 39 |
| **Audio streaming?** | 101 (33%) | 14 (35%) | 26 (30%) | 27 (34%) | 34 (33%) | 85 (25%) | 15 (22%) | 22 (27%) | 19 (22%) | 29 (30%) |
| (Missing) | 14 | 0 | 2 | 2 | 10 | 7 | 1 | 1 | 3 | 2 |
| *minutes per week* | 210 (70, 490) | 420 (210, 840) | 195 (60, 630) | 165 (60, 840) | 210 (80, 420) | 240 (105, 571) | 210 (120, 420) | 420 (175, 630) | 90 (60, 420) | 420 (161, 840) |
| (Missing) | 223 | 26 | 62 | 55 | 80 | 260 | 55 | 63 | 72 | 70 |
| **Gaming?** | 159 (51%) | 10 (25%) | 32 (37%) | 57 (71%) | 60 (58%) | 143 (43%) | 11 (16%) | 29 (36%) | 47 (53%) | 56 (58%) |
| (Missing) | 14 | 0 | 2 | 2 | 10 | 9 | 1 | 3 | 3 | 2 |
| *minutes per week* | 420 (180, 630) | 420 (180, 840) | 405 (130, 420) | 420 (138, 555) | 420 (210, 840) | 369 (120, 427) | 123 (30, 480) | 180 (70, 420) | 420 (168, 420) | 420 (210, 840) |
| (Missing) | 176 | 30 | 56 | 30 | 60 | 214 | 59 | 55 | 48 | 52 |
| **Cordless calling?** | 34 (11%) | 7 (18%) | 10 (12%) | 10 (13%) | 7 (6.9%) | 38 (11%) | 4 (5.8%) | 12 (15%) | 9 (10%) | 13 (13%) |
| (Missing) | 19 | 0 | 2 | 5 | 12 | 9 | 1 | 3 | 3 | 2 |
| *minutes per week* | 121 (60, 420) | 420 (70, 420) | 65 (30, 270) | 420 (120, 420) | 60 (5, 121) | 120 (30, 420) | 282 (45, 567) | 45 (15, 315) | 45 (15, 300) | 210 (120, 420) |
| (Missing) | 290 | 33 | 78 | 72 | 107 | 306 | 66 | 72 | 82 | 86 |
| *^1^*n (%); Median (Q1, Q3) | | | | | | | | | | |

Table C3. SP-CINT’s dataset summarized for child’s age and parent’s education

|  | **Parent's education No-University**, N = 180 | | | | **Parent's education University**, N = 226 | | | |
| --- | --- | --- | --- | --- | --- | --- | --- | --- |
| **Variables** | **Overall** N = 180*^1^* | **Age** **3–5**, n = 72*^1^* | **Age** **6–8**, n = 55*^1^* | **Age** **9–11**, n = 53*^1^* | **Overall** N = 226*^1^* | **Age** **3–5**, n = 82*^1^* | **Age** **6–8**, n = 63*^1^* | **Age** **9–11**, n = 81*^1^* |
| **Phone calling?** | 79 (44%) | 24 (33%) | 26 (47%) | 29 (55%) | 105 (46%) | 33 (40%) | 29 (46%) | 43 (53%) |
| (Missing) | 0 | 0 | 0 | 0 | 0 | 0 | 0 | 0 |
| *minutes per week* | 140 (35, 210) | 210 (105, 210) | 105 (28, 210) | 140 (18, 210) | 140 (0, 210) | 140 (70, 210) | 105 (0, 210) | 140 (32, 210) |
| (Missing) | 119 | 53 | 33 | 33 | 143 | 56 | 38 | 49 |
| **App calling?** | 67 (37%) | 16 (22%) | 23 (42%) | 28 (53%) | 96 (42%) | 31 (38%) | 25 (40%) | 40 (49%) |
| (Missing) | 0 | 0 | 0 | 0 | 0 | 0 | 0 | 0 |
| *minutes per week* | 210 (70, 210) | 210 (88, 210) | 210 (70, 210) | 210 (70, 210) | 210 (105, 210) | 158 (88, 210) | 210 (88, 210) | 210 (140, 217) |
| (Missing) | 143 | 60 | 41 | 42 | 173 | 62 | 51 | 60 |
| **Video calling?** | 77 (43%) | 29 (40%) | 26 (47%) | 22 (42%) | 97 (43%) | 40 (49%) | 24 (38%) | 33 (41%) |
| (Missing) | 0 | 0 | 0 | 0 | 0 | 0 | 0 | 0 |
| *minutes per week* | 210 (105, 210) | 210 (70, 210) | 210 (140, 210) | 210 (140, 210) | 140 (105, 210) | 105 (70, 210) | 140 (105, 210) | 210 (140, 210) |
| (Missing) | 131 | 49 | 41 | 41 | 166 | 55 | 50 | 61 |
| **Video streaming?** | 109 (61%) | 49 (68%) | 29 (53%) | 31 (58%) | 146 (65%) | 56 (68%) | 41 (65%) | 49 (60%) |
| (Missing) | 0 | 0 | 0 | 0 | 0 | 0 | 0 | 0 |
| *minutes per week* | 210 (210, 280) | 210 (210, 350) | 210 (210, 210) | 193 (105, 263) | 210 (210, 210) | 210 (175, 210) | 210 (140, 210) | 210 (210, 210) |
| (Missing) | 133 | 54 | 38 | 41 | 171 | 58 | 48 | 65 |
| **Audio streaming?** | 41 (23%) | 17 (24%) | 12 (22%) | 12 (23%) | 68 (30%) | 20 (24%) | 15 (24%) | 33 (41%) |
| (Missing) | 0 | 0 | 0 | 0 | 0 | 0 | 0 | 0 |
| *minutes per week* | 210 (210, 210) | 210 (210, 350) | 210 (210, 210) | 210 (105, 315) | 158 (88, 210) | 105 (70, 210) | 280 (210, 315) | 140 (105, 210) |
| (Missing) | 163 | 66 | 47 | 50 | 194 | 69 | 58 | 67 |
| **Gaming?** | 78 (43%) | 22 (31%) | 26 (47%) | 30 (57%) | 94 (42%) | 26 (32%) | 24 (38%) | 44 (54%) |
| (Missing) | 0 | 0 | 0 | 0 | 0 | 0 | 0 | 0 |
| *minutes per week* | 210 (168, 210) | 210 (210, 280) | 210 (105, 210) | 210 (210, 210) | 154 (70, 210) | 210 (140, 210) | 210 (70, 210) | 140 (70, 175) |
| (Missing) | 150 | 63 | 44 | 43 | 194 | 69 | 57 | 68 |
| **Cordless calling?** | 22 (12%) | 7 (9.7%) | 7 (13%) | 8 (15%) | 27 (12%) | 12 (15%) | 6 (9.5%) | 9 (11%) |
| (Missing) | 0 | 0 | 0 | 0 | 0 | 0 | 0 | 0 |
| *minutes per week* | 105 (0, 210) | 0 (0, 105) | 105 (35, 210) | 105 (35, 210) | 105 (70, 210) | 88 (53, 210) | 210 (70, 315) | 95 (70, 140) |
| (Missing) | 165 | 68 | 50 | 47 | 205 | 70 | 60 | 75 |
| *^1^*n (%); Median (Q1, Q3) | | | | | | | | |

Table D1. NL-QUALT’s dataset summarized for child’s age and level of urbanization

|  | **Urban level City**, N = 103 | | | | | **Urban level Town**, N = 333 | | | | | **Urban level Village**, N = 301 | | | | |
| --- | --- | --- | --- | --- | --- | --- | --- | --- | --- | --- | --- | --- | --- | --- | --- |
| **Variables** | **Overall** N = 103*^1^* | **Age** **0-2**, n = 30*^1^* | **Age** **3-5**, n = 36*^1^* | **Age** **6-8**, n = 25*^1^* | **Age** **9-11**, n = 12*^1^* | **Overall** N = 333*^1^* | **Age** **0-2**, n = 98*^1^* | **Age** **3-5**, n = 98*^1^* | **Age** **6-8**, n = 75*^1^* | **Age** **9-11**, n = 62*^1^* | **Overall** N = 301*^1^* | **Age** **0-2**, n = 84*^1^* | **Age** **3-5**, n = 90*^1^* | **Age** **6-8**, n = 75*^1^* | **Age** **9-11**, n = 52*^1^* |
| **Phone calling?** | 39 (38%) | 14 (47%) | 15 (42%) | 5 (20%) | 5 (42%) | 111 (33%) | 26 (27%) | 26 (27%) | 22 (29%) | 37 (60%) | 97 (32%) | 23 (27%) | 30 (33%) | 20 (27%) | 24 (46%) |
| (Missing) | 0 | 0 | 0 | 0 | 0 | 0 | 0 | 0 | 0 | 0 | 0 | 0 | 0 | 0 | 0 |
| *minutes per week* | 7 (3, 15) | 5 (3, 15) | 10 (5, 15) | 3 (3, 5) | 4 (3, 15) | 10 (5, 10) | 8 (5, 15) | 10 (4, 10) | 10 (5, 15) | 10 (5, 10) | 10 (5, 10) | 9 (5, 13) | 6 (5, 10) | 10 (5, 10) | 10 (10, 10) |
| (Missing) | 69 | 19 | 22 | 20 | 8 | 236 | 76 | 73 | 56 | 31 | 215 | 64 | 62 | 58 | 31 |
| **App calling?** | 15 (16%) | 1 (3.7%) | 5 (15%) | 4 (16%) | 5 (45%) | 43 (14%) | 3 (3.2%) | 16 (17%) | 5 (7.2%) | 19 (35%) | 48 (17%) | 8 (9.9%) | 10 (12%) | 10 (14%) | 20 (42%) |
| (Missing) | 7 | 3 | 3 | 0 | 1 | 21 | 5 | 2 | 6 | 8 | 16 | 3 | 5 | 4 | 4 |
| *minutes per week* | 5.0 (5.0, 10.0) | NA (NA, NA) | 5.0 (5.0, 10.0) | 10.0 (3.0, 10.0) | 5.0 (5.0, 10.0) | 5 (3, 10) | 3 (3, 10) | 8 (2, 10) | 5 (2, 5) | 5 (5, 15) | 5.0 (2.0, 10.0) | 5.0 (5.0, 10.0) | 3.5 (2.0, 5.0) | 4.0 (2.0, 10.0) | 10.0 (5.0, 15.0) |
| (Missing) | 92 | 30 | 33 | 22 | 7 | 298 | 95 | 88 | 70 | 45 | 262 | 77 | 82 | 68 | 35 |
| **Video calling?** | 31 (34%) | 8 (31%) | 12 (40%) | 5 (22%) | 6 (55%) | 91 (31%) | 23 (26%) | 38 (41%) | 11 (16%) | 19 (38%) | 86 (31%) | 19 (24%) | 35 (43%) | 11 (16%) | 21 (48%) |
| (Missing) | 13 | 4 | 6 | 2 | 1 | 35 | 10 | 6 | 7 | 12 | 27 | 4 | 8 | 7 | 8 |
| *minutes per week* | 10 (5, 15) | 13 (4, 25) | 10 (5, 15) | 5 (5, 10) | 8 (4, 13) | 10 (5, 20) | 10 (5, 30) | 10 (5, 15) | 10 (10, 15) | 15 (5, 30) | 10 (5, 12) | 10 (5, 20) | 5 (5, 10) | 10 (5, 20) | 10 (5, 20) |
| (Missing) | 74 | 22 | 25 | 19 | 8 | 246 | 76 | 62 | 63 | 45 | 220 | 69 | 56 | 64 | 31 |
| **Video streaming?** | 49 (56%) | 7 (27%) | 22 (76%) | 13 (54%) | 7 (78%) | 187 (64%) | 31 (36%) | 59 (67%) | 55 (81%) | 42 (89%) | 166 (62%) | 22 (29%) | 58 (73%) | 52 (76%) | 34 (77%) |
| (Missing) | 15 | 4 | 7 | 1 | 3 | 43 | 11 | 10 | 7 | 15 | 35 | 9 | 11 | 7 | 8 |
| *minutes per week* | 60 (25, 280) | 15 (15, 60) | 60 (20, 250) | 110 (60, 400) | 60 (30, 120) | 100 (30, 300) | 30 (20, 70) | 60 (30, 140) | 205 (60, 420) | 240 (60, 420) | 120 (60, 300) | 60 (10, 120) | 90 (30, 210) | 205 (60, 350) | 210 (120, 420) |
| (Missing) | 56 | 25 | 14 | 12 | 5 | 156 | 69 | 41 | 23 | 23 | 139 | 63 | 32 | 25 | 19 |
| **Audio streaming?** | 33 (38%) | 8 (32%) | 8 (28%) | 11 (48%) | 6 (67%) | 84 (30%) | 19 (23%) | 24 (28%) | 24 (37%) | 17 (38%) | 90 (34%) | 14 (19%) | 20 (25%) | 25 (38%) | 31 (70%) |
| (Missing) | 17 | 5 | 7 | 2 | 3 | 53 | 14 | 12 | 10 | 17 | 38 | 10 | 11 | 9 | 8 |
| *minutes per week* | 50 (23, 60) | 38 (8, 120) | 45 (30, 60) | 60 (30, 60) | 30 (10, 60) | 60 (30, 100) | 20 (10, 30) | 60 (30, 120) | 60 (50, 90) | 60 (30, 120) | 60 (30, 100) | 30 (20, 60) | 60 (30, 160) | 45 (30, 60) | 83 (50, 150) |
| (Missing) | 71 | 22 | 28 | 15 | 6 | 250 | 80 | 75 | 50 | 45 | 216 | 71 | 71 | 50 | 24 |
| **Gaming?** | 31 (36%) | 0 (0%) | 8 (28%) | 14 (61%) | 9 (100%) | 92 (33%) | 2 (2.4%) | 16 (18%) | 38 (58%) | 36 (86%) | 90 (35%) | 0 (0%) | 18 (23%) | 37 (57%) | 35 (88%) |
| (Missing) | 17 | 5 | 7 | 2 | 3 | 55 | 15 | 11 | 9 | 20 | 47 | 13 | 12 | 10 | 12 |
| *minutes per week* | 60 (30, 210) | NA (NA, NA) | 45 (25, 60) | 60 (60, 120) | 210 (200, 300) | 100 (40, 240) | 35 (10, 60) | 30 (30, 100) | 70 (30, 230) | 120 (100, 300) | 70 (30, 200) | NA (NA, NA) | 30 (15, 60) | 60 (45, 150) | 165 (60, 300) |
| (Missing) | 72 | 30 | 28 | 11 | 3 | 248 | 96 | 83 | 40 | 29 | 214 | 84 | 74 | 38 | 18 |
| **Cordless calling?** | 1 (1.2%) | 0 (0%) | 0 (0%) | 0 (0%) | 1 (11%) | 5 (1.8%) | 0 (0%) | 1 (1.2%) | 2 (3.2%) | 2 (4.9%) | 7 (2.8%) | 1 (1.4%) | 0 (0%) | 2 (3.1%) | 4 (11%) |
| (Missing) | 19 | 6 | 8 | 2 | 3 | 61 | 16 | 12 | 12 | 21 | 51 | 13 | 14 | 10 | 14 |
| *minutes per week* | 0 (0, 0) | 0 (0, 0) | NA (NA, NA) | NA (NA, NA) | 0 (0, 0) | 2.0 (0.0, 5.0) | 0.0 (0.0, 0.0) | 20.0 (20.0, 20.0) | 2.0 (2.0, 2.0) | 2.5 (0.0, 5.0) | 3.0 (1.0, 5.0) | 2.5 (2.5, 2.5) | 0.0 (0.0, 0.0) | 1.0 (0.0, 3.0) | 5.0 (5.0, 10.0) |
| (Missing) | 101 | 29 | 36 | 25 | 11 | 328 | 97 | 97 | 74 | 60 | 292 | 83 | 89 | 72 | 48 |
| *^1^*n (%); Median (Q1, Q3) | | | | | | | | | | | | | | | |

Table D2. NL-CINT’s dataset summarized for child’s age and level of urbanisation

|  | **Urban level City**, N = 274 | | | | | **Urban level Town**, N = 274 | | | | | **Urban level Village**, N = 120 | | | | |
| --- | --- | --- | --- | --- | --- | --- | --- | --- | --- | --- | --- | --- | --- | --- | --- |
| **Variables** | **Overall** N = 274*^1^* | **Age** **0-2**, n = 55*^1^* | **Age** **3-5**, n = 66*^1^* | **Age** **6-8**, n = 67*^1^* | **Age** **9-11**, n = 86*^1^* | **Overall** N = 274*^1^* | **Age** **0-2**, n = 40*^1^* | **Age** **3-5**, n = 77*^1^* | **Age** **6-8**, n = 78*^1^* | **Age** **9-11**, n = 79*^1^* | **Overall** N = 120*^1^* | **Age** **0-2**, n = 15*^1^* | **Age** **3-5**, n = 29*^1^* | **Age** **6-8**, n = 28*^1^* | **Age** **9-11**, n = 48*^1^* |
| **Phone calling?** | 178 (65%) | 25 (45%) | 32 (48%) | 46 (69%) | 75 (87%) | 167 (61%) | 17 (43%) | 41 (53%) | 46 (59%) | 63 (80%) | 58 (48%) | 2 (13%) | 13 (45%) | 10 (36%) | 33 (69%) |
| (Missing) | 0 | 0 | 0 | 0 | 0 | 0 | 0 | 0 | 0 | 0 | 0 | 0 | 0 | 0 | 0 |
| *minutes per week* | 121 (60, 420) | 60 (20, 210) | 120 (60, 420) | 105 (60, 420) | 205 (105, 550) | 70 (30, 420) | 90 (12, 735) | 70 (30, 190) | 70 (30, 240) | 75 (40, 420) | 60 (30, 140) | 70 (20, 120) | 60 (20, 60) | 130 (30, 175) | 67 (53, 415) |
| (Missing) | 96 | 30 | 34 | 21 | 11 | 109 | 24 | 36 | 33 | 16 | 63 | 13 | 16 | 18 | 16 |
| **App calling?** | 143 (53%) | 21 (38%) | 29 (44%) | 41 (62%) | 52 (63%) | 133 (49%) | 14 (36%) | 23 (30%) | 45 (58%) | 51 (65%) | 59 (50%) | 3 (20%) | 12 (41%) | 10 (36%) | 34 (76%) |
| (Missing) | 5 | 0 | 0 | 1 | 4 | 2 | 1 | 0 | 1 | 0 | 3 | 0 | 0 | 0 | 3 |
| *minutes per week* | 105 (60, 420) | 90 (30, 420) | 70 (60, 420) | 105 (35, 240) | 180 (105, 473) | 90 (30, 210) | 105 (60, 210) | 90 (30, 420) | 90 (30, 420) | 90 (35, 210) | 60 (30, 120) | 10 (4, 840) | 75 (5, 263) | 122 (70, 240) | 60 (30, 70) |
| (Missing) | 131 | 34 | 37 | 26 | 34 | 141 | 26 | 54 | 33 | 28 | 61 | 12 | 17 | 18 | 14 |
| **Video calling?** | 129 (48%) | 24 (44%) | 27 (41%) | 32 (48%) | 46 (58%) | 146 (54%) | 21 (54%) | 35 (45%) | 44 (57%) | 46 (58%) | 64 (55%) | 5 (33%) | 18 (62%) | 11 (39%) | 30 (67%) |
| (Missing) | 7 | 0 | 0 | 1 | 6 | 2 | 1 | 0 | 1 | 0 | 3 | 0 | 0 | 0 | 3 |
| *minutes per week* | 105 (60, 300) | 73 (33, 145) | 70 (35, 180) | 70 (30, 315) | 220 (70, 420) | 60 (30, 150) | 60 (20, 72) | 30 (15, 140) | 60 (30, 210) | 66 (30, 210) | 60 (20, 135) | 20 (15, 80) | 60 (15, 60) | 30 (20, 364) | 80 (30, 280) |
| (Missing) | 147 | 31 | 40 | 36 | 40 | 128 | 19 | 42 | 34 | 33 | 56 | 10 | 11 | 17 | 18 |
| **Video streaming?** | 149 (56%) | 20 (36%) | 48 (74%) | 40 (62%) | 41 (51%) | 182 (67%) | 24 (62%) | 54 (71%) | 53 (69%) | 51 (65%) | 71 (61%) | 8 (53%) | 19 (66%) | 17 (61%) | 27 (60%) |
| (Missing) | 9 | 0 | 1 | 2 | 6 | 3 | 1 | 1 | 1 | 0 | 3 | 0 | 0 | 0 | 3 |
| *minutes per week* | 420 (210, 840) | 420 (262, 840) | 308 (130, 630) | 454 (273, 840) | 420 (300, 1,162) | 420 (210, 840) | 368 (138, 420) | 420 (150, 630) | 420 (210, 637) | 420 (240, 840) | 420 (180, 630) | 750 (330, 1,260) | 315 (120, 420) | 240 (120, 420) | 420 (180, 840) |
| (Missing) | 125 | 35 | 18 | 27 | 45 | 92 | 16 | 23 | 25 | 28 | 49 | 7 | 10 | 11 | 21 |
| **Audio streaming?** | 80 (31%) | 15 (27%) | 19 (30%) | 20 (31%) | 26 (33%) | 73 (27%) | 9 (23%) | 23 (30%) | 19 (25%) | 22 (28%) | 33 (29%) | 5 (33%) | 6 (21%) | 7 (26%) | 15 (34%) |
| (Missing) | 12 | 0 | 2 | 2 | 8 | 4 | 1 | 1 | 2 | 0 | 5 | 0 | 0 | 1 | 4 |
| *minutes per week* | 420 (120, 840) | 210 (150, 420) | 420 (90, 630) | 270 (45, 840) | 420 (210, 840) | 210 (60, 420) | 240 (120, 420) | 210 (60, 630) | 90 (60, 420) | 190 (105, 420) | 180 (60, 525) | 616 (420, 840) | 298 (35, 525) | 60 (60, 840) | 180 (60, 350) |
| (Missing) | 195 | 40 | 48 | 47 | 60 | 201 | 31 | 54 | 59 | 57 | 87 | 10 | 23 | 21 | 33 |
| **Gaming?** | 100 (38%) | 9 (16%) | 25 (40%) | 33 (51%) | 33 (42%) | 141 (52%) | 9 (23%) | 24 (32%) | 53 (70%) | 55 (70%) | 61 (53%) | 3 (20%) | 12 (41%) | 18 (67%) | 28 (64%) |
| (Missing) | 14 | 0 | 4 | 2 | 8 | 4 | 1 | 1 | 2 | 0 | 5 | 0 | 0 | 1 | 4 |
| *minutes per week* | 420 (210, 630) | 180 (60, 480) | 420 (315, 420) | 420 (210, 720) | 420 (360, 854) | 330 (140, 420) | 210 (60, 427) | 210 (120, 420) | 420 (175, 420) | 323 (175, 420) | 420 (98, 645) | 420 (20, 560) | 88 (45, 300) | 180 (60, 420) | 420 (300, 840) |
| (Missing) | 179 | 46 | 41 | 35 | 57 | 147 | 31 | 53 | 30 | 33 | 64 | 12 | 17 | 13 | 22 |
| **Cordless calling?** | 33 (13%) | 5 (9.1%) | 8 (13%) | 5 (7.7%) | 15 (19%) | 28 (10%) | 4 (10%) | 11 (14%) | 10 (13%) | 3 (3.8%) | 11 (9.9%) | 2 (13%) | 3 (10%) | 4 (16%) | 2 (4.8%) |
| (Missing) | 14 | 0 | 4 | 2 | 8 | 5 | 1 | 1 | 3 | 0 | 9 | 0 | 0 | 3 | 6 |
| *minutes per week* | 122 (60, 420) | 60 (60, 70) | 60 (30, 210) | 420 (300, 420) | 210 (120, 420) | 100 (20, 420) | 420 (271, 462) | 80 (20, 270) | 120 (20, 455) | 5 (5, 60) | 121 (30, 630) | 735 (630, 840) | 15 (5, 980) | 100 (45, 280) | 76 (30, 121) |
| (Missing) | 241 | 50 | 58 | 62 | 71 | 246 | 36 | 66 | 68 | 76 | 109 | 13 | 26 | 24 | 46 |
| *^1^*n (%); Median (Q1, Q3) | | | | | | | | | | | | | | | |

Table D3. SP-CINT´s dataset summarized for child’s age and level of urbanization

|  | **Urban level City**, N = 207 | | | | **Urban level Town**, N = 148 | | | | **Urban level Village**, N = 51 | | | | |
| --- | --- | --- | --- | --- | --- | --- | --- | --- | --- | --- | --- | --- | --- |
| **Variables** | **Overall** N = 207*^1^* | **Age** **3–5**, n = 68*^1^* | **Age** **6–8**, n = 63*^1^* | **Age** **9–11**, n = 76*^1^* | **Overall** N = 148*^1^* | **Age** **3–5**, n = 59*^1^* | **Age** **6–8**, n = 42*^1^* | **Age** **9–11**, n = 47*^1^* | | **Overall** N = 51*^1^* | **Age** **3–5**, n = 27*^1^* | **Age** **6–8**, n = 13*^1^* | **Age** **9–11**, n = 11*^1^* |
| **Phone calling?** | 96 (46%) | 26 (38%) | 27 (43%) | 43 (57%) | 67 (45%) | 21 (36%) | 20 (48%) | 26 (55%) | | 21 (41%) | 10 (37%) | 8 (62%) | 3 (27%) |
| (Missing) | 0 | 0 | 0 | 0 | 0 | 0 | 0 | 0 | | 0 | 0 | 0 | 0 |
| *minutes per week* | 140 (0, 210) | 210 (70, 210) | 105 (0, 210) | 140 (0, 210) | 140 (35, 210) | 140 (0, 210) | 88 (14, 210) | 140 (105, 210) | | 105 (53, 210) | 210 (105, 210) | 105 (28, 140) | 123 (35, 210) |
| (Missing) | 130 | 49 | 41 | 40 | 97 | 40 | 24 | 33 | | 35 | 20 | 6 | 9 |
| **App calling?** | 95 (46%) | 25 (37%) | 29 (46%) | 41 (54%) | 53 (36%) | 15 (25%) | 14 (33%) | 24 (51%) | | 15 (29%) | 7 (26%) | 5 (38%) | 3 (27%) |
| (Missing) | 0 | 0 | 0 | 0 | 0 | 0 | 0 | 0 | | 0 | 0 | 0 | 0 |
| *minutes per week* | 210 (105, 210) | 175 (105, 210) | 210 (70, 210) | 210 (140, 217) | 210 (140, 210) | 210 (140, 273) | 210 (140, 210) | 210 (140, 210) | | 112 (35, 210) | 70 (35, 210) | 182 (91, 210) | NA (NA, NA) |
| (Missing) | 155 | 53 | 48 | 54 | 120 | 48 | 35 | 37 | | 41 | 21 | 9 | 11 |
| **Video calling?** | 98 (47%) | 35 (51%) | 27 (43%) | 36 (47%) | 58 (39%) | 23 (39%) | 17 (40%) | 18 (38%) | | 18 (35%) | 11 (41%) | 6 (46%) | 1 (9.1%) |
| (Missing) | 0 | 0 | 0 | 0 | 0 | 0 | 0 | 0 | | 0 | 0 | 0 | 0 |
| *minutes per week* | 210 (105, 210) | 123 (70, 210) | 158 (119, 210) | 210 (140, 210) | 175 (105, 210) | 175 (70, 273) | 210 (88, 210) | 140 (140, 210) | | 140 (70, 210) | 105 (70, 210) | 140 (28, 210) | 175 (175, 175) |
| (Missing) | 152 | 46 | 51 | 55 | 107 | 40 | 30 | 37 | | 38 | 18 | 10 | 10 |
| **Video streaming?** | 130 (63%) | 50 (74%) | 38 (60%) | 42 (55%) | 91 (61%) | 34 (58%) | 27 (64%) | 30 (64%) | | 34 (67%) | 21 (78%) | 5 (38%) | 8 (73%) |
| (Missing) | 0 | 0 | 0 | 0 | 0 | 0 | 0 | 0 | | 0 | 0 | 0 | 0 |
| *minutes per week* | 210 (193, 210) | 210 (210, 210) | 210 (210, 217) | 210 (175, 210) | 210 (210, 210) | 210 (210, 315) | 210 (210, 210) | 210 (140, 210) | | 210 (140, 210) | 210 (140, 210) | 210 (210, 245) | 210 (105, 350) |
| (Missing) | 155 | 51 | 46 | 58 | 115 | 44 | 31 | 40 | | 34 | 17 | 9 | 8 |
| **Audio streaming?** | 63 (30%) | 15 (22%) | 18 (29%) | 30 (39%) | 33 (22%) | 13 (22%) | 7 (17%) | 13 (28%) | | 13 (25%) | 9 (33%) | 2 (15%) | 2 (18%) |
| (Missing) | 0 | 0 | 0 | 0 | 0 | 0 | 0 | 0 | | 0 | 0 | 0 | 0 |
| *minutes per week* | 210 (119, 210) | 210 (210, 315) | 210 (158, 245) | 175 (105, 210) | 158 (70, 263) | 70 (70, 315) | 210 (210, 263) | 88 (70, 105) | | 140 (70, 210) | 70 (70, 210) | 210 (210, 210) | NA (NA, NA) |
| (Missing) | 174 | 58 | 55 | 61 | 136 | 53 | 38 | 45 | | 47 | 24 | 12 | 11 |
| **Gaming?** | 95 (46%) | 25 (37%) | 28 (44%) | 42 (55%) | 64 (43%) | 16 (27%) | 20 (48%) | 28 (60%) | | 13 (25%) | 7 (26%) | 2 (15%) | 4 (36%) |
| (Missing) | 0 | 0 | 0 | 0 | 0 | 0 | 0 | 0 | | 0 | 0 | 0 | 0 |
| *minutes per week* | 210 (105, 210) | 210 (210, 280) | 210 (70, 280) | 140 (105, 210) | 210 (105, 210) | 210 (123, 263) | 210 (105, 210) | 210 (210, 280) | | 210 (140, 210) | 147 (140, 210) | 210 (210, 210) | 210 (210, 210) |
| (Missing) | 174 | 59 | 56 | 59 | 126 | 51 | 33 | 42 | | 44 | 22 | 12 | 10 |
| **Cordless calling?** | 28 (14%) | 12 (18%) | 9 (14%) | 7 (9.2%) | 15 (10%) | 5 (8.5%) | 3 (7.1%) | 7 (15%) | | 6 (12%) | 2 (7.4%) | 1 (7.7%) | 3 (27%) |
| (Missing) | 0 | 0 | 0 | 0 | 0 | 0 | 0 | 0 | | 0 | 0 | 0 | 0 |
| *minutes per week* | 105 (7, 210) | 70 (0, 210) | 158 (70, 315) | 105 (7, 210) | 70 (35, 105) | 70 (21, 105) | 210 (210, 210) | 70 (35, 105) | | 105 (70, 210) | 140 (70, 210) | 35 (35, 35) | 158 (105, 210) |
| (Missing) | 185 | 59 | 57 | 69 | 139 | 54 | 41 | 44 | | 46 | 25 | 12 | 9 |
| *^1^*n (%); Median (Q1, Q3) | | | | | | | | | | | | | |

*SM3 Cluster description*

Table E1. Description of clusters emerged from NL-QUALT dataset

| **Cluster** | **N** |  | **Child age** |  | **Phone call use** | **App call use** | **Video call use** | **Video stream use** | **Audio stream use** | **Gaming use** | **Phone call duration** | **App call duration** | **Video call duration** | **Video stream duration** | **Audio stream duration** | **Gaming duration** |
| --- | --- | --- | --- | --- | --- | --- | --- | --- | --- | --- | --- | --- | --- | --- | --- | --- |
| **NL_Q** | 740 |  | 3-5 | Boy | No | No | No | Yes | No | No | 0 | 0 | 0 | 30 | 0 | 0 |
| **NL_Q_0-2a** | 378 |  | 0-2 | Boy | No | No | No | Yes | No | No | 0 | 0 | 0 | 5 | 0 | 0 |
| **NL_Q_6-8** | 129 |  | 6-8 | Boy | No | No | No | Yes | No | Yes | 0 | 0 | 0 | 210 | 0 | 60 |
| **NL_Q_9-11** | 56 |  | 9-11 | Boy | Yes | Yes | Yes | Yes | Yes | Yes | 10 | 5 | 10 | 120 | 25 | 95 |
| **NL_Q_0-2b** | 177 |  | 0-2 | Girl | Yes | No | No | Yes | No | No | 5 | 0 | 0 | 30 | 0 | 0 |

Notes: “NL_Q” represents descriptives for the whole NL-QUALT dataset, the following rows describe the different clusters, each identified with their majoritarian age group and a letter to distinguish them when necessary. Mother educational level, Urban level, Parent’s age, Cordless use and Cordless duration have been excluded from the table because they are constant between clusters.

Table E2. Description of clusters emerged from NL-CINT dataset

| **Cluster** | **N** | **Child age** | **Child’s sex** | **Parent’s education** | **Urban level** | **Parent’s age** | **Phone call use** | **App call use** | **Videocall use** | **Video stream use** | **Audio stream use** | **Gaming use** | **Phone call duration** | **App call duration** | **Videocall duration** | **Video stream duration** | **Audio stream duration** | **Gaming duration** |
| --- | --- | --- | --- | --- | --- | --- | --- | --- | --- | --- | --- | --- | --- | --- | --- | --- | --- | --- |
| **NL_C** | 668 | 9-11 | Boy | University | City | <30 | Yes | Yes | Yes | Yes | No | No | 30 | 5 | 5 | 150 | 0 | 0 |
| **NL_C _0-2** | 187 | 0-2 | Boy | University | City | <30 | No | No | No | Yes | No | No | 0 | 0 | 0 | 30 | 0 | 0 |
| **NL_C _9-11a** | 164 | 9-11 | Girl | Non-University | Town | <30 | Yes | Yes | Yes | No | No | No | 115 | 87 | 6 | 0 | 0 | 0 |
| **NL_C _3-5** | 190 | 3-5 | Girl | Non-University | Town | 30<40 | No | No | No | Yes | No | Yes | 0 | 0 | 0 | 210 | 0 | 60 |
| **NL_C _9-11b** | 127 | 9-11 | Boy | University | City | <30 | Yes | Yes | Yes | Yes | Yes | Yes | 120 | 105 | 70 | 420 | 70 | 420 |

Note: “NL_C” represents descriptives for the whole Dutch CINT dataset, the following rows describe the different clusters, each identified with their majoritarian age group and a letter to distinguish them when necessary. Cordless use, Cordless duration have been excluded from the table because they are constant between clusters (all “0”).

Table E3. Description of clusters emerged from SP-CINT dataset

| **Cluster** | **N** | **Child age** | **Child´s sex** | **Parent’s age** | **Parent’s education** | **Urban level** | **Phone call use** | **App call use** | **Videocall use** | **Video stream use** | **Audio stream use** | **Gaming use** | **Phone call duration** | **App call duration** | **Videocall duration** |
| --- | --- | --- | --- | --- | --- | --- | --- | --- | --- | --- | --- | --- | --- | --- | --- |
| **SP_C** | 406 | 3-5 | Girl | 30<40 | University | City | No | No | No | Yes | No | No | 0 | 0 | 0 |
| **SP_C _3-5a** | 102 | 3-5 | Girl | 30<40 | Non-university | Town | No | No | No | Yes | No | No | 0 | 0 | 0 |
| **SP_C _3-5b** | 68 | 3-5 | Girl | <30 | University | City | Yes | Yes | Yes | Yes | No | No | 0 | 0 | 0 |
| **SP_C_9-11a** | 101 | 9-11 | Girl | 30<40 | University | City | No | No | No | No | No | No | 0 | 0 | 0 |
| **SP_C_9-11b** | 70 | 9-11 | Boy | 30<40 | University | City | Yes | Yes | Yes | Yes | Yes | Yes | 87.5 | 87.5 | 0 |
| **SP_C_9-11c** | 65 | 9-11 | Girl | 30<40 | Non-university | Town | Yes | Yes | Yes | Yes | No | Yes | 105 | 0 | 35 |

Note: “SP_C” represents descriptives for the whole Spanish dataset, the following rows describe the different clusters, each identified with their majoritarian age group and a letter to distinguish them when necessary. Cordless use, Cordless duration, Gaming duration, Audio stream duration, and Video stream duration been excluded from the table because they are constant between clusters (all “0”).

Figure F1: Hierarchical cluster analysis of mobile device use in the NL-QUALT dataset.

Figure F2: Hierarchical cluster analysis of mobile device use in the NL-CINT dataset.

Figure F3: Hierarchical cluster analysis of mobile device use in the SP-CINT dataset.

SM4 Interaction of rules and age on the performance of screen-related activities

Table G. Coefficients of the interaction term in the regression models (glm(dependent variable ~ rules * child age)).

| Dependent Variable | Dataset | Regression Model | Coefficient (β) | 95% CI |
| --- | --- | --- | --- | --- |
| Videogaming use | NL-Qualtrics | Logistic | 0.12 | (-0.05, 0.31) |
| Videostreaming use | NL-Qualtrics | Logistic | 0.16* | (0.04, 0.29) |
| Videocalling use | NL-Qualtrics | Logistic | 0.05 | (-0.04, 0.15) |
| Videogaming duration | NL-Qualtrics | Linear | 3.58 | (-14.10, 21.26) |
| Videostreaming duration | NL-Qualtrics | Linear | -16.19* | (-30.30, -2.08) |
| Videocalling duration | NL-Qualtrics | Linear | -1.08* | (-2.01, -0.16) |
| Videogaming use | NL-CINT | Logistic | 0.02 | (-0.07, 0.12) |
| Videostreaming use | NL-CINT | Logistic | 0.07 | (-0.02, 0.16) |
| Videocalling use | NL-CINT | Logistic | 0.06 | (-0.03, 0.15) |
| Videogaming duration | NL-CINT | Linear | 0.91 | (-26.73, 28.55) |
| Videostreaming duration | NL-CINT | Linear | 10.55 | (-16.58, 37.67) |
| Videocalling duration | NL-CINT | Linear | 5.80 | (-7.05, 18.65) |
| Videogaming use | SP-CINT | Logistic | 0.10 | (-0.06, 0.27) |
| Videostreaming use | SP-CINT | Logistic | 0.07 | (-0.10, 0.24) |
| Videocalling use | SP-CINT | Logistic | -0.03 | (-0.18, 0.12) |
| Videogaming duration | SP-CINT | Linear | 5.41 | (-15.56, 26.3) |
| Videostreaming duration | SP-CINT | Linear | -1.49 | (-28.66, 25.66) |
| Videocalling duration | SP-CINT | Linear | 9.81 | (-5.39, 25.02) |

1. The following question was repeated for each activity, namely *make or receive calls (using a cellular network), make or receive voice calls via an app (e.g. WhatsApp, Hangouts, Viber, Skype), video calling via an app (e.g. WhatsApp, Hangouts, Viber, Skype), stream videos online (e.g. Netflix, YouTube, Apple TV), stream online audio (music, audiobooks) (e.g. Spotify, Tidal, online radio, online podcasts), play online games, use a cordless home phone at least once a week to make or receive calls (cordless home phones are portable handsets designed for use with a landline).*
2. This question was repeated for each of the devices participants selected in the previous question, namely smartphone, tablet, and laptop
